# The Mitochondrial Ca^2+^ Uniporter Complex (MCUC) of Trypanosoma brucei Is a Hetero-oligomer That Contains Novel Subunits Essential for Ca^2+^ Uptake

**DOI:** 10.1128/mBio.01700-18

**Published:** 2018-09-18

**Authors:** Guozhong Huang, Roberto Docampo

**Affiliations:** a Center for Tropical and Emerging Global Diseases, University of Georgia, Athens, Georgia, USA; b Department of Cellular Biology, University of Georgia, Athens, Georgia, USA; Washington University School of Medicine

**Keywords:** *Trypanosoma brucei*, calcium uniporter, mitochondria

## Abstract

The mitochondrial calcium uniporter complex (MCUC) is a highly selective channel that conducts calcium ions across the organelle inner membrane. We previously characterized Trypanosoma brucei’s MCU (TbMCU) as an essential component of the MCUC required for parasite viability and infectivity. In this study, we characterize its paralog T. brucei MCUb (TbMCUb) and report the identification of two novel components of the complex that we named TbMCUc and TbMCUd. These new MCUC proteins are unique and conserved only in trypanosomatids. *In situ* tagging and immunofluorescence microscopy revealed that they colocalize with TbMCU and TbMCUb to the mitochondria of T. brucei. Blue Native PAGE and immunodetection analyses indicated that the MCUC proteins exist in a large protein complex with a molecular weight of approximately 380 kDa. RNA interference (RNAi) or overexpression of the TbMCUc and TbMCUd genes significantly reduced or enhanced mitochondrial Ca^2+^ uptake in T. brucei, respectively, without affecting the mitochondrial membrane potential, indicating that they are essential components of the MCUC of this parasite. The specific interactions of TbMCU with TbMCUb, TbMCUc, or TbMCUd were confirmed by coimmunoprecipitation and split-ubiquitin membrane-based yeast two-hybrid (MYTH) assays. Furthermore, combining mutagenesis analysis with MYTH assays revealed that transmembrane helices (TMHs) were determinant of the interactions between TbMCUC subunits. In summary, our study has identified two novel essential components of the MCUC of T. brucei and defined their direct physical interactions with the other subunits that result in a hetero-oligomeric MCUC.

## Abstract (precis)

Trypanosoma brucei causes human African trypanosomiasis and nagana in animals. The finding of a mitochondrial calcium uniporter (MCU) conserved in this parasite was essential for the discovery of the gene encoding the pore subunit. Mitochondrial Ca^2+^ transport mediated by the MUC complex is critical in Trypanosoma brucei for shaping the dynamics of cytosolic Ca^2+^ increases, for the bioenergetics of the cells, and for viability and infectivity. We found that one component of the complex (MCUb) does not act as a dominant negative effector of the channel as in vertebrate cells and that the TbMCUC possesses two unique subunits (MCUc and MCUd) present only in trypanosomatids and required for Ca^2+^ transport. The study of the interactions between these four subunits (MCU, MCUb, MCUc, and MCUd) by a variety of techniques that include coimmunoprecipitation, split-ubiquitin membrane-based yeast two-hybrid assays, and site-directed mutagenesis suggests that they interact through their transmembrane helices to form hetero-oligomers.

## INTRODUCTION

The Trypanosoma brucei group of parasites causes nagana in cattle and African trypanosomiasis or sleeping sickness in humans. Two of the best-studied life cycle stages of T. brucei are the procyclic form (PCF), which is found in the tsetse fly vector, and the bloodstream form (BSF), which is present in the blood of infected animal hosts. Although both stages have a single mitochondrion, the PCF mitochondrion has a respiratory chain, while the BSF mitochondrion does not possess a functional respiratory chain or oxidative phosphorylation. BSF trypanosomes rely on the reverse action of the ATP synthase to maintain a mitochondrial membrane potential (ΔΨ_m_) (1–4), which is required for protein (5) and Ca^2+^ (2) uptake. Both stages have a functional mitochondrial Ca^2+^ uniporter (MCU) (6, 7), which is essential for growth and infectivity (7).

The finding of a MCU in trypanosomatids with characteristics similar to that present in mammalian cells (8, 9) was important for the discovery of the molecular nature of a modulator of the channel, mitochondrial calcium uptake 1 (MICU1) (10), and the pore subunit of the uniporter or MCU (11–13). After this significant discovery, other subunits of the MCU complex (MCUC), such as MCU regulator 1 (MCUR1) (14), MICU2 and MICU3 (15), MCUb (16), and essential MCU regulator (EMRE) (17), were described in mammals. Trypanosomatids lack orthologs to EMRE, MCUR1, and MICU3 (18). Current models of the metazoan uniporter indicate that MCU spans the mitochondrial inner membrane forming the pore and is surrounded by the other regulatory subunits as a large complex (11, 17). Interestingly, the recombinant pore domains of MCU from Caenorhabditis elegans (19) form homo-pentamers, while those from several fungi, like Neurospora crassa (20), Neosartorya fischeri (21), Metarhizium acridum (22), Fusarium graminearum (22), and Cyphellophora europaea (23), and those from zebra fish (23) form homo-tetramers *in vitro*. However, which is the oligomeric state *in vivo* and how MCU interacts with its membrane partners MCUb and EMRE remain to be investigated.

In this work, we report that T. brucei MCUC (TbMCUC) has two additional subunits not found in mammalian cells that we have named TbMCUc and TbMCUd. These subunits are essential for mitochondrial Ca^2+^ uptake, and their physical interaction with the other subunits (TbMCU and TbMCUb) of the MCUC was studied by coimmunoprecipitation and split-ubiquitin membrane-based yeast two-hybrid (MYTH) assays. The results suggest that the MCUC is a hetero-oligomer which is composed of four distinct pore-forming and Ca^2+^-conducting subunits (TbMCU, TbMCUb, TbMCUc, and TbMCUd). This is the first report in which the oligomeric state of a hetero-MCU complex has been defined using MYTH technology.

## RESULTS

### Identification of novel subunits of the TbMCUC.

**FIG 1:**
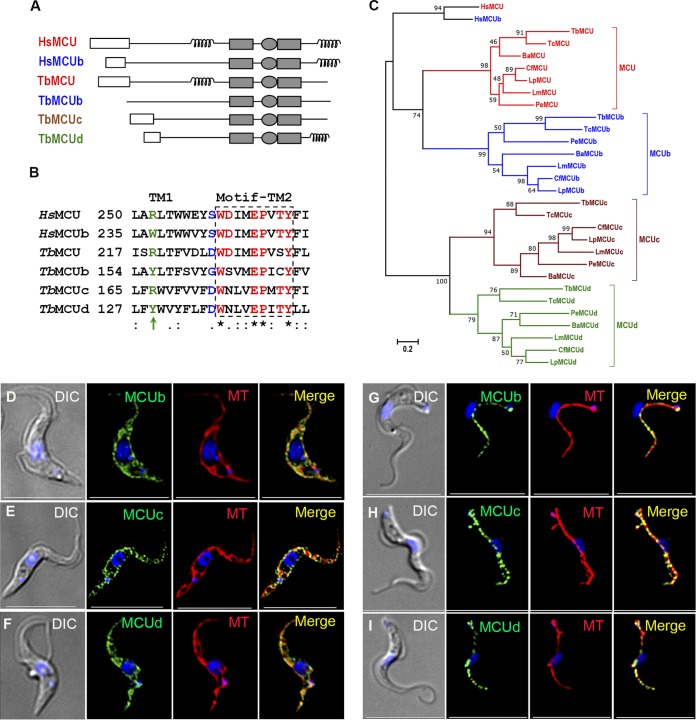
Domain architecture, alignment, phylogeny, and localization of TbMCUC subunits. (A) Comparison of the protein domain architectures of TbMCUC subunits with Homo sapiens MCU (HsMCU) and MCUb. The mitochondrial targeting sequence (MTS; white rectangle), coiled-coil domain (coil), transmembrane domains (TM; shaded rectangles), and conserved WDXXEPXTY motif (shaded ellipse) are shown. (B) Alignment of the C-terminal parts of the first transmembrane domain (TM1) and conserved WDXXEPXTY motif near or in the N-terminal parts of the second transmembrane domain (TM2) (boxed with a dashed line) of TbMCUC subunits with human MCU and MCUb. One critical conserved substitution, R to W/Y, in the TM1 domains is shown in green and indicated with an arrow. The Ru360-sensitive residue substitution S to D/G in the pore region is shown in blue. Conserved residues in the WDXXEPXTY motif are shown in red. The symbols “*,” “:,” and “.” represent identical, conserved, and semiconserved amino acid substitutions, respectively. (C) Phylogenetic tree of trypanosomatid and human MCUC subunits. The detailed information for species and accession numbers in TriTrypDB or GenBank is described in the legend of Fig. S1 in the supplemental material. The scale bar corresponds to a distance of 20 changes per 100 amino acid positions. Ba, Blechomonas ayalai; Cf, Crithidia fasciculata; Lp, Leptomonas pyrrhocoris; Lm, Leishmania major; Pe, Phytomonas sp. isolate EM1. (D to F) smFLAG-tagged TbMCUb (D), smHA-tagged TbMCUc (E), and smV5-tagged TbMCUd (F) colocalized with MitoTracker (MT) to the mitochondria of PCF trypanosomes (Pearson’s correlation coefficients [PCC], 0.8459, 0.8425, and 0.8901, respectively). (G to I) smV5-tagged TbMCUb (G), smV5-tagged TbMCUc (H), and smHA-tagged TbMCUd (I) colocalized with MT to the mitochondria of BSF trypanosomes (PCC of 0.6645, 0.87626, and 0.6894, respectively). Yellow in merged images indicates colocalization. DIC, differential interference contrast microscopy. Scale bars, 10 µm.

To screen for genes encoding TbMCU orthologs, the amino acid sequence of TbMCU (TriTryp Database accession number Tb427tmp.47.0014) was used to search TriTrypDB using BLASTp. This search yielded three putative proteins, each with two transmembrane domains, that we designated TbMCUb (Tb427.10.300) (18), TbMCUc (Tb427tmp.02.1760), and TbMCUd (Tb427.10.2150), which have high identity to TbMCU (25 to 31% identity in 62 to 191 amino acids). The open reading frames predict proteins of 254, 249, and 214 amino acids, with apparent molecular weights of 28.4, 27.8, and 24.7 kDa, respectively, for TbMCUb, TbMCUc, and TbMCUd. Interestingly, TbMCUc and TbMCUd, along with TbMCUb, exhibited 16 to 19% overall identity and 28 to 34% similarity with TbMCU and contained most of the conserved domains of TbMCU, including two transmembrane domains and one modified putative Ca^2+^ selectivity filter, and belong to the MCU family (Pfam: PF04678). Most of them contain a putative mitochondrial targeting signal (MTS), with the exception of TbMCUb, and some have coiled-coil motifs (Fig. 1A). TbMCUb does not contain a typical N-terminal MTS, as predicted by MitoProt (Fig. 1A and see Fig. S1A in the supplemental material), but it has a possible cleavage site for a signal sequence between amino acids 51 and 52, as predicted by PSORT II. To search for any MCU orthologs in other organisms, the amino acid sequences of TbMCU, TbMCUb, TbMCUc, TbMCUd, and human MCU and MCUb were used to search TriTrypDB and GenBank using BLASTp (iterative PSI-BLASTp). TbMCU and TbMCUb orthologs are widely distributed among most eukaryotes, but those of TbMCUc and TbMCUd are found only in trypanosomatids (Fig. 1C and Fig. S1B), including the free-living trypanosomatid Bodo saltans, which has orthologs to MCU (BS46740), MCUb (BS74080), and MCUc (BS20060) but not to MCUd. Significantly, the WDXXEPXTY motif found in the pore region of eukaryotic MCU and MCUb was WNXXEPXTY in both MCUc and MCUd in almost all trypanosomatid species examined (Fig. 1B and Fig. S1B). The serine (S) residue responsible for the sensitivity to Ru360 in the pore region of mammalian MCUs (11) was changed to D/G in the trypanosomatid MCU paralogs (Fig. 1B and Fig. S1B). The critical conserved substitution R to W between MCU and MCUb near the pore of mammalian MCUb (16) was instead R to Y in trypanosomatid MCUb orthologs (Fig. S1B). Additionally, 11 of 28 MCU orthologs from trypanosomatids did not have a typical N-terminal MTS, as predicted by MitoProt (Fig. S1A).

10.1128/mBio.01700-18.1 FIG S1 Domain architecture and alignment of trypanosomatid MCUC subunits. (A) Protein domain architecture of 28 trypanosomatid MCUC subunits with human MCU and MCUb. MTS (white rectangle), mitochondrial targeting sequence; coil, coiled-coil domain; TM (shaded rectangle), transmembrane domain; WDXXEPXTY (shaded ellipse), the conserved sequence motif of the MCU pore forming a putative Ca^2+^ selectivity filter. (B) Amino acid sequence alignment of the transmembrane domains (TM1 and TM2) and Ca^2+^ selectivity filter region of 28 trypanosomatid MCUC proteins with human MCU and MCUb. The TriTrypDB and GenBank accession numbers for 30 MCUC subunits from the following human and trypanosomatids are as follows: Homo sapiens (HsMCU, NP_612366.1; HsMCUb, NP_060388.2), T. brucei (TbMCU, Tb427tmp.47.0014; TbMCUb, Tb427.10.300; TbMCUc, Tb427tmp.02.1760; TbMCUd, Tb427.10.2150), T. cruzi (TcMCU, TcCLB.503893.120; TcMCUb, TcCLB.504069.4; TcMCUc, TcCLB.506177.110; TcMCUd; TcCLB.511367.330), Blechomonas ayalai (BaMCU, Baya_054_0020; BaMCUb, Baya_017_0740; BaMCUc, Baya_012_0180; BaMCUd, Baya_025_0400), Crithidia fasciculata (CfMCU, CFAC1_230043800; CfMCUb, CFAC1_130026800; CfMCUc, CFAC1_220034400; CfMCUd, CFAC1_260009600), Leptomonas pyrrhocoris (LpMCU, LpyrH10_07_1010; LpMCUb, LpyrH10_14_2200; LpMCUc, LpyrH10_20_1340; LpMCUd, LpyrH10_14_0410), Leishmania major (LmMCU, LmjF.27.0780; LmMCUb, LmjF.21.1690; LmMCUc, LmjF.13.0600; LmMCUd, LmjF.21.0350), and *Phytomonas* sp. isolate EM1 (PeMCU, NCBI Protein Database accession number CCW60994.1; PeMCUb, CCW62912.1; PeMCUc, CCW63803.1; PeMCUd, CCW61886.1). Download FIG S1, JPG file, 1.5 MB. Copyright © 2018 Huang and Docampo. 2018 Huang and Docampo This is an open-access article distributed under the terms of the Creative Commons Attribution 4.0 International license.

### Localization of TbMCUb, TbMCUc, and TbMCUd to the mitochondria of PCF and BSF trypanosomes.

**FIG 2:**
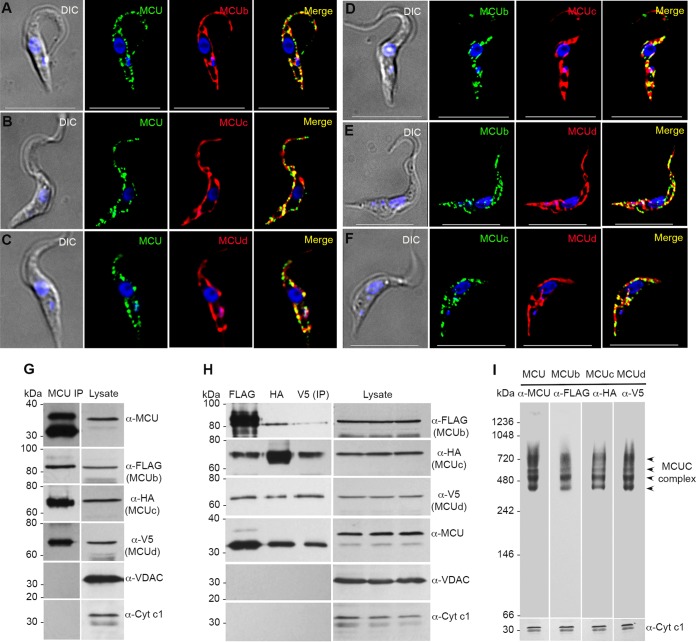
Colocalization and coimmunoprecipitation of TbMCU, TbMCUb, TbMCUc, and TbMCUd using the triple-smFP-tagged TbMCUC PCF cell line. (A to C) Colocalization of smFLAG-tagged TbMCUb (A), smHA-tagged TbMCUc (B), and smV5-tagged TbMCUd (C) with TbMCU (PCC of 0.7602, 0.7003, and 0.7774, respectively). (D to E) Colocalization of smFLAG-tagged TbMCUb with smHA-tagged TbMCUc (D) or smV5-tagged TbMCUd (E) (PCC of 0.6596 and 0.6305, respectively). DIC, differential interference contrast microscopy. Scale bars = 10 μm. (F) Colocalization of smHA-tagged TbMCUc with smV5-tagged TbMCUd (PCC of 0.7144). Scale bars, 10 µm. The merged images indicate colocalization (in yellow). (G) Coimmunoprecipitation of TbMCUb, TbMCUc, and TbMCUd with TbMCU. Cell lysates from the triple-smFP-tagged TbMCUC cell line were incubated with anti-TbMCU antibody, immunoprecipitates (IP) were resolved by SDS-PAGE, and input lysates and immunoprecipitates were blotted with antibodies against TbMCU, FLAG, HA, V5, TbVDAC, and TbCyt *c*_1_. (H) Anti-FLAG, anti-HA, and anti-V5 immunoprecipitations were performed using lysates from the triple-smFP-tagged TbMCUC cell line, and input lysates and immunoprecipitates were blotted with the antibodies as described for panel G. (I) BN-PAGE analyses of crude PCF mitochondrial vesicles from the triple-smFP-tagged TbMCUC cell line. Immunoblot analyses were performed using antibodies against TbMCU, FLAG, HA, and V5. Antibodies against TbCyt *c*_1_ were used as a loading control but detected on a SDS-PAGE gel. Arrowheads indicate one dominant band at ∼500 kDa and three weak bands at 420 to 720 kDa. Multiple bands probably reflected different combinations of tagged and nontagged subunits in the complex, since only one allele of each endogenous gene was generally tagged. Markers are shown on the left, and the antibodies used in immunoblots are shown on the right.

To investigate the localization of the novel MCUC subunits, the C terminus of each protein was tagged in PCF trypanosomes with a hemagglutinin (HA) tag using homologous recombination with the endogenous gene locus. However, we were unable to detect the proteins by Western blot or immunofluorescence analyses, probably as a result of their low expression. We then generated cell lines overexpressing each gene tagged with HA, as described in Materials and Methods. All these proteins colocalized with MitoTracker to the mitochondria of PCF trypanosomes (Fig. S2A to C). Western blot analysis detected bands of the expected size in the overexpressing cell lines that were not visible in the absence of tetracycline induction (Fig. S2D). To confirm the localization of these proteins, we modified the pMOTag vectors for *in situ* epitope tagging (24) and tagged the C terminus of each endogenous protein with a high-performance tag (spaghetti monster fluorescent protein [smFP]) with FLAG, HA, or V5 epitope tags (Fig. S2E) that has recently been described to enhance the detection of weakly expressed proteins (25–27). Figure 1D to I show that all the proteins, along with TbMCU (Fig. S2F), colocalize with MitoTracker (MT) to the mitochondria of PCF (Fig. 1D to F) and BSF (Fig. 1G to I) trypanosomes. Western blot analyses confirmed the expression of the proteins with the expected apparent molecular weights (Fig. S2G and Fig. 2G and H).

10.1128/mBio.01700-18.2 FIG S2 Localization of overexpressing TbMCUb, TbMCUc, or TbMCUd and smFP-tagged TbMCU to the mitochondria of PCF trypanosomes. (A to C) HA-tagged overexpressing TbMCUb, TbMCUc, or TbMCUd colocalized with MitoTracker (MT) to the mitochondria of T. brucei (Pearson’s correlation coefficients of 0.8676, 0.8158, and 0.8452, respectively). The merged images indicate colocalization (in yellow). DIC, differential interference contrast microscopy. Scale bars = 10 µm. (D) Western blot analysis of tetracycline-inducible TbMCUb-HA, TbMCUc-HA, and TbMCU-HA genes overexpressed in PCF trypanosomes using monoclonal antibodies against HA. MagicMark XP was used as a molecular weight marker, and bands corresponding to TbMCUb, TbMCUc, and TbMCUd are shown. The same amount of proteins of non-tetracycline-induced (–Tet) lysates was loaded as a control. Tubulin was used as a loading control (bottom panel). (E) Map of modified pMOTag vectors for *in situ* spaghetti monster epitope tagging. The plasmids were modified from pMOTag vectors (24) using spaghetti monster fluorescent proteins (smFPs) with epitope tags (FLAG, HA, or V5) (25) as described in Materials and Methods. (F and G) Immunofluorescence and Western blot analyses of smHA-tagged TbMCU. (F) smHA-tagged TbMCU colocalized with MT to the mitochondria of PCF trypanosomes (Pearson’s correlation coefficient of 0.7740). The merged images indicate colocalization (in yellow). DIC, differential interference contrast microscopy. Scale bars = 10 µm. (G) Tagging with smHA was confirmed by Western blot analysis of TbMCU using anti-HA antibodies. MagicMark XP marker (Invitrogen) is at the left side, and the corresponding band of smHA-tagged TbMCU is shown. Tubulin was used as a loading control (bottom panel). Download FIG S2, JPG file, 0.8 MB. Copyright © 2018 Huang and Docampo. 2018 Huang and Docampo This is an open-access article distributed under the terms of the Creative Commons Attribution 4.0 International license.

### Colocalization and coimmunoprecipitation of TbMCU, TbMCUb, TbMCUc, and TbMCUd.

To investigate whether TbMCU can form hetero-oligomers in T. brucei, we generated a single PCF transgenic cell line in which TbMCUb, TbMCUc, and TbMCUd were endogenously C-terminally tagged with smFLAG, smHA, and smV5, respectively. Immunofluorescence analyses of the triple-smFP-tagged transgenic cell line revealed that TbMCUb, TbMCUc, and TbMCUd colocalized with the TbMCU to the mitochondria of PCF trypanosomes, using anti-TbMCU (α-TbMCU), α-FLAG, α-HA and/or α-V5 antibodies, respectively (Fig. 2A to F). The cells were lysed, and immunoprecipitations were done with α-TbMCU, α-FLAG, α-HA, or α-V5 antibodies, respectively. TbMCUb, TbMCUc, and TbMCUd, but not T. brucei voltage-dependent anion channel (TbVDAC) or cytochrome *c*_1_ (TbCyt *c*_1_), which are localized to the outer and inner mitochondrial membranes, respectively, were detected by protein immunoblotting after immunoprecipitation of TbMCU from the triple-smFP-tagged *T. brucei* PCF cell line (Fig. 2G). Similarly, TbMCU, TbMCUc, and TbMCUd were pulled down with TbMCUb using α-FLAG; TbMCU, TbMCUb, and TbMCUd were pulled down with TbMCUc using α-HA; and also TbMCU, TbMCUb, and TbMCUc were pulled down with TbMCUd using α-V5, but neither TbVDAC nor TbCyt *c*_1_ was immunoprecipitated with any of the antibodies (Fig. 2H). To confirm that these TbMCUC subunits belong to the same large protein complex, crude PCF mitochondrial vesicles were isolated from the triple-smFP-tagged TbMCUC cell line and lysed with dodecylmaltoside (DDM) for blue Native PAGE (BN-PAGE) and then immunoblotted with α-TbMCU, α-FLAG, α-HA, or α-V5 antibodies, respectively. TbMCU, TbMCUb, TbMCUc, and TbMCUd migrated with the same separation pattern, with one dominant band at ∼500 kDa and three weak bands ranging from 420 to 720 kDa (Fig. 2I), suggesting that they exist in a large protein complex with a net molecular weight of approximately 380 kDa after removal of the triple smFP tags. Hence, the results suggest that the TbMCU complex is a hetero-oligomer containing at least 4 subunits: TbMCU, TbMCUb, TbMCUc, and TbMCUd.

### RNAi and overexpression of new TbMCUC subunits affect mitochondrial Ca^2+^ uptake but do not alter ΔΨ_m_.

**FIG 3:**
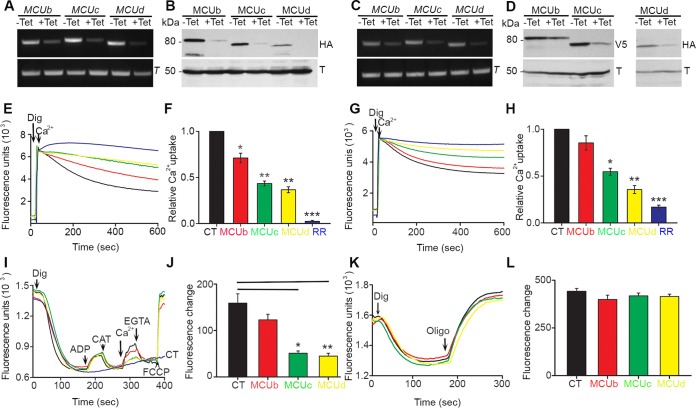
RNAi silencing of TbMCUC subunits reduces Ca^2+^ uptake of digitonin-permeabilized PCF and BSF trypanosomes. (A and C) RT-PCR analyses of TbMCUb, TbMCUc, or TbMCUd gene RNAi of PCF (A) and BSF (C) trypanosomes grown in the absence (–Tet) or presence (+Tet) of tetracycline. RT-PCR analysis of tubulin (T) is shown as a control. (B and D) Western blot analyses of TbMCUb, TbMCUc, and TbMCUd gene RNAi of PCF (B) and BSF (D) trypanosomes grown in the absence or presence of tetracycline. Total lysates (30 μg) were subjected to 10% SDS–PAGE before transfer to a nitrocellulose membrane and then stained with antibodies against HA or V5 (top). Bands of the sizes expected for smFP-tagged TbMCUb (∼80 kDa), TbMCUc (64 kDa), and TbMCUd (62 or 67 kDa) were detected in T. brucei lysates. Membranes were stripped and reincubated with antibody against tubulin as a loading control (bottom). (E) PCF trypanosomes (5 × 10^7^ cells) were added to the standard reaction buffer (2.45 ml) containing 2 mM succinate, 10 μM EGTA, and 0.5 μM calcium green-5N, and the reaction was started with digitonin (Dig, 50 μM). (G) BSF trypanosomes (2 × 10^8^ cells) were added to the standard reaction buffer (1.95 ml) containing 1 mM ATP, sodium orthovanadate (500 μM), 10 μM EGTA, and 0.5 μM calcium green-5N, and the reaction was started with digitonin (40 μM). CaCl_2_ (20 μM) and ruthenium red (RR; 16 μM) were added where indicated. (F and H) Relative Ca^2+^ uptake at 600 s compared with that of control trypanosomes grown in the absence of tetracycline, considered 1.0 (control [CT]). Values are means ± standard deviations. *n* = 3; *, *P*  < 0.05; **, *P*  < 0.01; ***, *P*  < 0.001 (Student's *t* test). (I) PCF trypanosomes (5 × 10^7^ cells) were added to the standard reaction buffer (2.45 ml) containing 2 mM succinate and 5 μM Safranin O, and the reaction was started with digitonin (50 μM). ADP (10 μM), carboxyatractyloside (CAT, 2 μg/ml), CaCl_2_ (20 μM), EGTA (200 μM), and FCCP (10 μM) were added where indicated. (J) Changes in Safranin O fluorescence after addition of Ca^2+^ to PCF trypanosomes with and without Tet. Values are means ± standard deviations. *n =* 3; * or **, *P < *0.01 (Student's *t* test). (K) BSF trypanosomes (2 × 10^8^ cells) were added to the standard reaction buffer (1.95 ml) containing EGTA (20 μM), ATP (1 mM), sodium orthovanadate (500 μM), and 12.5 μM Safranin O. The reaction was started with digitonin (40 μM). (L) Changes in Safranin O fluorescence after addition of oligomycin (Oligo; 2.5 μg/ml) to BSF trypanosomes with and without *Tet*. Values are means ± standard deviations. *n = 3*. Different colors of fluorescence traces were black (for the control [CT]), red (TbMCUb gene RNAi), green (TbMCUc gene RNAi), yellow (TbMCUd gene RNAi), and blue (RR).

We used RNA interference (RNAi) to knock down the expression of each protein, which, in contrast to the results reported with TbMCU (7), did not result in growth defects in either PCF (Fig. S3A) or BSF (Fig. S3C) trypanosomes. However, a significant growth defect was observed when PCF TbMCUc and TbMCUd mutants were grown in a glucose-deficient medium (SDM-80) (31) (Fig. S3D). Relative reverse transcription-PCR (RT-PCR) and ImageJ analyses showed that the mRNA was downregulated by 52 to 79% after 4 days of tetracycline addition to PCF trypanosomes (Fig. 3A). Similar results were obtained using BSF trypanosomes (Fig. 3C). To confirm the protein downregulation by RNAi, we tagged each of the novel TbMCUC subunits at the C terminus of each endogenous protein with a high-performance smFP epitope using the corresponding TbMCUb, TbMCUc, or TbMCUd gene RNAi cell line of PCF and BSF trypanosomes. Western blot analyses revealed a correlative decrease by 76 to 91% of TbMCUc and TbMCUd in both PCF (Fig. 3B) and BSF (Fig. 3D) trypanosomes. However, TbMCUb had only an 18% reduction (Fig. 3D). Further phenotypic analyses were done after 2 days and 4 days of growth for BSF and PCF trypanosomes, respectively.

10.1128/mBio.01700-18.3 FIG S3 Growth of PCF with TbMCUb, TbMCUc, and TbMCUd genes downregulated by RNAi and of overexpressing cell lines. Growth curves of PCF (A, B, D) and BSF (C) trypanosomes in the absence (–Tet; black lines) or presence (TbMCUb gene RNAi [red], TbMCUc gene RNAi [green], and TbMCUd gene RNAi [yellow]) of 1 µg/ml tetracycline in SDM-79 (A and B), SDM-80 (D), or HMI-9 (C) medium for the indicated numbers of days. Values are means ± standard deviations (*n* = 3). *, *P* < 0.05; ns, not significant (Student’s *t* test). Download FIG S3, JPG file, 0.6 MB. Copyright © 2018 Huang and Docampo. 2018 Huang and Docampo This is an open-access article distributed under the terms of the Creative Commons Attribution 4.0 International license.

To investigate the ability of the knockdown cell lines to take up Ca^2+^, we monitored Ca^2+^ uptake with calcium green-5N in digitonin-permeabilized PCF and BSF trypanosomes. A decrease in fluorescence indicates decreasing medium Ca^2+^ or increasing vesicular Ca^2+^. Figure 3 shows that addition of 50 µM digitonin in the presence of 5 mM succinate in the case of PCF trypanosomes (Fig. 3E and F) or 1 mM ATP in the case of BSF trypanosomes (Fig. 3G and H) and 20 µM Ca^2+^ produced a fast decrease in Ca^2+^ concentration starting after a period of about 30 s. This Ca^2+^ uptake activity was fully eliminated by the addition of 16 µM ruthenium red, indicating that it is due to the uniporter. Knockdown of TbMCUc and TbMCUd significantly decreased the mitochondria’s ability to take up Ca^2+^ in both PCF and BSF trypanosomes (Fig. 3E to H), while knockdown of TbMCUb only significantly decreased uptake in PCF trypanosomes, which is consistent with the weak effect of RNAi on protein expression in BSF trypanosomes (Fig. 3D).

**FIG 4:**
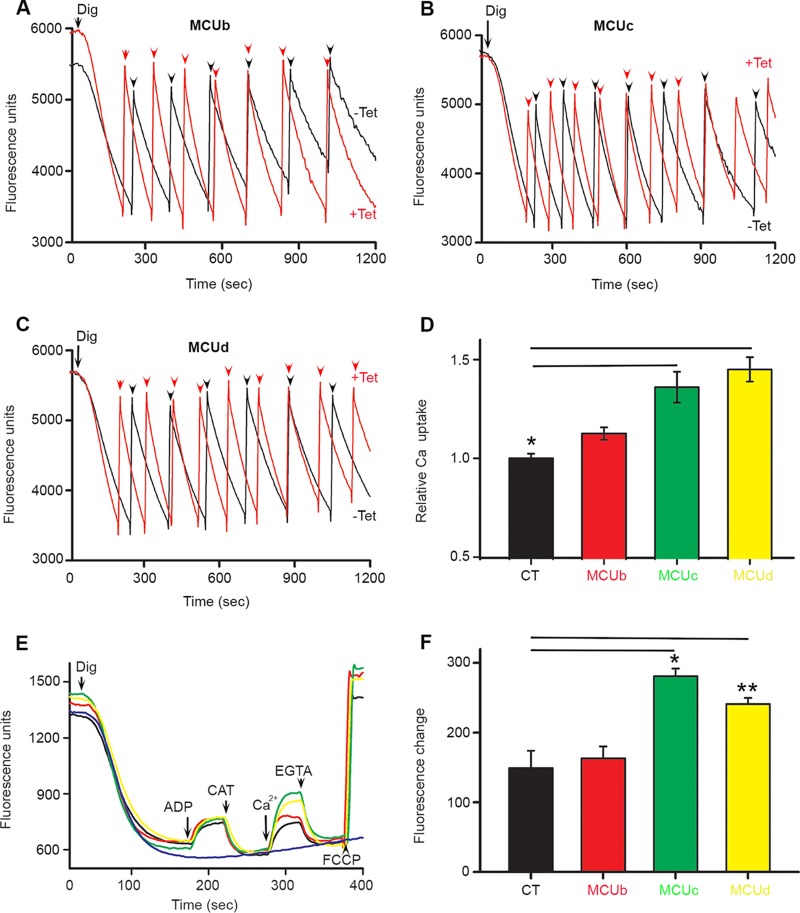
Overexpression of TbMCUC subunits increases mitochondrial Ca^2+^ uptake but does not alter mitochondrial membrane potential (ΔΨ_m_). (A to C) Ca^2+^ uptake by digitonin-permeabilized PCF trypanosomes overexpressing the TbMCUb, TbMCUc, or TbMCUd gene, respectively. PCF trypanosomes (5 × 10^7^ cells) were added to the standard reaction buffer (2.45 ml) containing 2 mM succinate and 0.5 μM calcium green-5N, and the reaction was started with digitonin (50 μM). Multiple pulses of CaCl_2_ (arrowheads) were added to a final concentration of 8 μM. A decrease in fluorescence indicates decreasing medium Ca^2+^ or increasing vesicular Ca^2+^. (D) Relative Ca^2+^ uptake in PCF trypanosomes in which the TbMCUb, TbMCUc, or TbMCUd gene is overexpressed (with Tet) 1,200 s after additions of Ca^2+^, compared to that in uninduced trypanosomes (considered 1) (without Tet). Values are means ± standard deviations. *n* = 3; *, *P* < 0.01 (Student's *t* test). (E) PCF trypanosomes (5 × 10^7^ cells) were added to the standard reaction buffer (2.45 ml) containing 2 mM succinate and 5 μM Safranin O, and the reaction was started with digitonin (50 μM). ADP (10 μM), carboxyatractyloside (2 μg/ml), CaCl_2_ (20 μM), EGTA (200 μM), and FCCP (10 μM) were added where indicated. (F) Changes in Safranin O fluorescence after addition of Ca^2+^ to PCF trypanosomes with and without *Tet*. Values are means ± standard deviations. *n* = 3; *, *P* < 0.05, or **, *P* < 0.01 (Student's *t* test). Different colors of fluorescence traces were black (CT), red (overexpressed TbMCUb), green (overexpressed TbMCUc), and yellow (overexpressed TbMCUd).

The mitochondria of permeabilized control PCF trypanosomes were capable of buffering multiple pulses of exogenously added Ca^2+^, and overexpression of the TbMCUc or TbMCUd gene increased significantly the ability of their mitochondria to accumulate Ca^2+^ in response to Ca^2+^ pulses (Fig. 4). The lack of a dominant negative effect of the overexpressed TbMCUb gene (Fig. 4A and 4D) is consistent with results reported for T. cruzi (28) and different from those reported for HeLa cells (16).

To investigate whether down- or upregulated expression of the new TbMCUC subunits affects ΔΨ_m_, we used Safranin O to measure ΔΨ_m_ in digitonin-permeabilized PCF trypanosomes in the presence of succinate as the mitochondrial substrate. When Safranin O was used, an increase in fluorescence after addition of digitonin indicates stacking of the dye to the energized inner mitochondrial membrane (Fig. 3I and 4E). Addition of ADP induced a small decrease in membrane potential, indicating ADP phosphorylation. ΔΨ_m_ returned to its initial level after addition of the mitochondrial ADP/ATP carrier inhibitor carboxyatractyloside (CAT) (Fig. 3I and Fig. 4E). Ca^2+^ uptake was also observed in BSF in the presence of ATP (Fig. 3K). Addition of the uncoupler carbonyl cyanide 4-(trifluoromethoxy) phenylhydrazone (FCCP) in PCF (Fig. 3I and 4E) or the ATP synthase inhibitor oligomycin in BSF (Fig. 3K) collapsed the membrane potential. Knockdown (Fig. S3A and C) or overexpression (Fig. S3B) of the TbMCUb, TbMCUc, or TbMCUd gene, which does not affect growth, altered only mitochondrial Ca^2+^ uptake but did not affect the ΔΨ_m_ at the steady state or ADP phosphorylation (Fig. 3I and J and Fig. 4E and F). Collectively, the newly identified TbMCUC subunits have the same properties of TbMCU (7) and other characterized eukaryotic MCUs (11, 12) upon Ca^2+^ uptake and ΔΨ_m_.

### Physical direct interactions between TbMCUC subunits.

**FIG 5:**
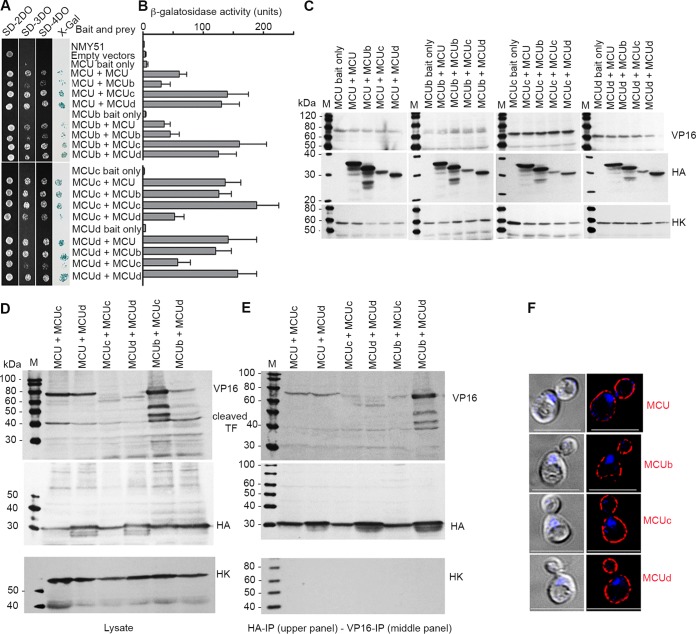
Verification of direct physical interactions among TbMCUC subunits by split-ubiquitin membrane-based Y2H (MYTH) assays and coimmunoprecipitation. (A) Growth of yeast reporter strain NMY51 expressing the bait (TbMCU, TbMCUb, TbMCUc, or TbMCUd) together with the prey (TbMCU, TbMCUb, TbMCUc, or TbMCUd) on SD selection agar plates (SD-2DO, SD-3DO, SD-4DO, and SD-4DO plus X-Gal). Exponentially growing cells were harvested and resuspended at 5 × 10^6^ cells/ml, and 3 µl of a 1:10 dilution was spotted on an SD agar plate and incubated at 30°C for 2 to 3 days. Blue colonies growing on high-stringency SD-4DO plus X-Gal plates confirmed the interactions between TbMCUC subunits. The reporter NMY51 strain harboring the empty bait (pBT3-SUC) and prey (pPR3N) vectors or TbMCUC subunit bait only in pBT3-SUC were used as negative controls. (B) A quantitative β-galactosidase (β-Gal) activity assay determines the interaction strength of TbMCUC subunits. The yeast NMY51 strain coexpressing TbMCUC bait and prey as described for panel A was used in a β-Gal microplate assay using *o*-nitrophenyl-β-d-galactopyranoside (ONPG) as the substrate. Each column represents a mean ± standard deviation (*n* = 3; 8 colonies for each independent experiment). (C) Immunoblot validation of TbMCUC baits or bait-prey pairs (as indicated) expressed in the yeast NMY51 stain. Lysates containing 60 µg of protein from yeast transformants were subjected to SDS-PAGE on 10% polyacrylamide gels and transferred to a nitrocellulose membrane. The blots were probed with antibodies against VP16 (for bait), HA (for prey), and hexokinase (HK; for the loading control). Lanes M, molecular markers. (D and E) Coimmunoprecipitation validation of interactions between TbMCUC subunits as indicated. Yeast lysates from the transformants were incubated with anti-VP16 or anti-HA antibodies, immunoprecipitates were resolved by SDS-PAGE, and input lysates (D) and immunoprecipitates (E) were blotted with antibodies against VP16, HA, and hexokinase. Immunoblotting of the input lysates confirmed both bait expression and TF cleavage (as indicated). Hexokinase was used as a negative control. (F) Fluorescence microscopy images validated proper yeast plasma membrane localization of expressing bait TbMCU-, TbMCUb-, TbMCUc-, or TbMCUd-Cub-LexA-VP16. Scale bars = 5 µm. Left images were obtained by DIC.

To better understand the possible organization of TbMCUC subunits in the hetero-oligomeric complex of T. brucei, we used the split-ubiquitin membrane-based yeast two-hybrid (MYTH) assays (29) (Fig. S4A) to determine the physical direct interactions between TbMCUC subunits in Saccharomyces cerevisiae. The split-ubiquitin system allows detection of *in vivo* interaction between membrane proteins that have their N and/or C terminus located in the cytosol (Fig. S4A). The membrane topology of TbMCU, TbMCUb, TbMCUc, and TbMCUd predicted with Protter showed that these membrane proteins could be localized to the yeast plasma membrane with both their N and C termini facing the cytosol (Fig. S5A). In the MYTH assays, TbMCU, TbMCUb, TbMCUc, or TbMCUd (the bait, without the mitochondrial targeting signal [MTS]) was fused to the C-terminal half of ubiquitin (C_ub_) and the artificial transcription factor LexA-VP16 (TF). TbMCU, TbMCUb, TbMCUc, or TbMCUd (the prey, without the MTS) was fused to the mutated half of ubiquitin (N_ub_G), and the interaction of the protein partners was monitored by the release of the TF, which translocates to the nucleus, where it binds to LexA operators situated upstream of reporter genes (*HIS3*, *ADE2*, *lacZ*) via its Lex DNA binding domain. The reporter genes enable the yeast to grow on defined media lacking histidine or/and adenine, while *lacZ* encodes the enzyme β-galactosidase (β-Gal), resulting in the growth of yeast in selective medium and color development in β-galactosidase assays. Figure 5F showed that each of the TbMCUC subunits was expressed as a bait, with the yeast beta-fructofuranosidase or invertase (SUCrose 2 [SUC]) signal sequence instead of the MTS targeted correctly to the yeast plasma membrane. The yeast reporter strain expressing the bait TbMCU, TbMCUb, TbMCUc, or TbMCUd alone or with the empty prey vector did not grow on the selective synthetic dropout (SD) medium plates (SD medium with a triple dropout [SD-3DO], SD-4DO, and SD-4DO plus X-Gal [5-bromo-4-chloro-3-indolyl-β-d-galactopyranoside]) (Fig. 5A), indicating that the baits were not self-activated. The strain expressing TbMCU as a bait and TbMCUc or TbMCUd as a prey enabled growth on the high-stringency selective SD-4DO plates and had high β-galactosidase activities (Fig. 5A and B), suggesting that TbMCU interacted strongly with TbMCUc and TbMCUd, respectively. Similarly, MYTH screens identified that TbMCUb interacted strongly with TbMCUc and TbMCUd and that TbMCUc or TbMCUd also strongly interacted with itself (Fig. 5A and B). However, MYTH screens showed that TbMCU interacted weakly with TbMCUb, that TbMCUc interacted weakly with TbMCUd, and that TbMCU or TbMCUb interacted weakly with itself (Fig. 5A and B). Expression of each of the baits or the bait-prey pairs in yeast was confirmed by Western blot analyses using antitag antibodies from the MYTH expression vectors pBT3-SUC and pPR3N (29), i.e., antibodies α-VP16 for the bait and antibodies α-HA for the prey (Fig. 5C). To eliminate false-positive interactions, the specific interactions among the TbMCUC subunits were confirmed by bait-prey swapping (Fig. 5A and B), coimmunoprecipitations (Fig. 5D and E), and immunofluorescence subcellular colocalization (Fig. S4C and D). The interaction strength of TbMCUC subunits in yeast may reflect the relative proximity of the subunits in a large protein complex; thus, our data suggest a hetero-hexameric model for the putative organization and composition of the TbMCU complex in T. brucei (Fig. S4B).

10.1128/mBio.01700-18.4 FIG S4 Physical interactions between TbMCU, TbMCUb, TbMCUc, and TbMCUd. (A) The scheme depicts the MYTH system used for analysis of TbMCUC interactions. The bait must be a membrane protein that is tagged with the C terminus of ubiquitin (Cub) fused to a transcription factor (LexA-VP16). The prey protein can be soluble or membrane bound and is fused to the N terminus of ubiquitin (NubG). The interaction of bait and prey protein reconstituted a full-size pseudoubiquitin molecule, which is recognized by cytosolic deubiquitinating enzymes (UBPs). Subsequently, the enzyme cleaves off the transcription factor LexA-VP16. LexA-VP16 enters the nucleus and activates the expression of reporter genes (*HIS3, ADE*, and *lacZ*). Therefore, the cells containing interacting bait-prey pairs can grow on synthetic dropout (SD) media. (B) The scheme depicts the putative organization and composition of a hetero-hexameric TbMCU complex. (C and D) Immunofluorescence validation of colocalization of TbMCUC interaction pairs (as indicated) to the yeast plasma membrane. The baits TbMCU, TbMCUb, TbMCUc, and TbMCUd tagged with LexA-VP16 and the preys TbMCUc and TbMCUd tagged with HA were stained with antibodies against VP16 (red) and HA (green), respectively. The PCC of the bait-prey pairs TbMCU-TbMCUc, TbMCU-TbMCUd, TbMCUc-TbMCUc, TbMCUd-TbMCUd, TbMCUb-TbMCUc, and TbMCUb-TbMCUd are 0.7360, 0.7027, 0.6964, 0.6802, 0.6491, and 0.6031, respectively. Left images were obtained by DIC microscopy. Scale bars = 5 µm. The merged images indicate colocalization (in yellow). Download FIG S4, JPG file, 1.1 MB. Copyright © 2018 Huang and Docampo. 2018 Huang and Docampo This is an open-access article distributed under the terms of the Creative Commons Attribution 4.0 International license.

10.1128/mBio.01700-18.5 FIG S5 TbMCUC subunit topology models and MYTH constructs. (A) Putative topology models of TbMCUC subunits predicted with Protter. Putative mitochondrial targeting sequences (MTS) of TbMCU, TbMCUc, and TbMCUd, predicted by MitoProt, are marked in red. TbMCUb does not have a typical MTS. (B) MYTH constructs generated. (C) Alignment of TM domains and the conserved WDXXEPXTY sequence motif of TbMCUC subunits. The highly conserved amino acid residues in the TMH1 and TMH2 of TbMCUC subunits are highlighted by arrows and were replaced in the mutants as indicated. Download FIG S5, JPG file, 1.1 MB. Copyright © 2018 Huang and Docampo. 2018 Huang and Docampo This is an open-access article distributed under the terms of the Creative Commons Attribution 4.0 International license.

### TMHs are determinant of the interactions between TbMCUC subunits.

**FIG 6:**
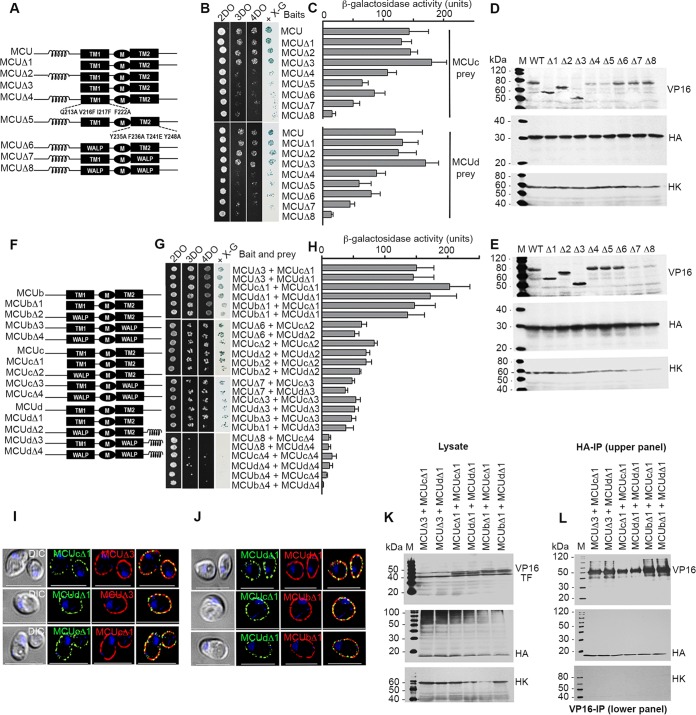
Determination of specific interactions between the transmembrane domains of TbMCUC subunits by mutagenesis, MYTH analyses, and coimmunoprecipitation. (A) The scheme depicts wild-type TbMCU and truncated and substitution mutant constructs. Coil, coiled-coil domain; TM1 and TM2 (black rectangles), transmembrane domains 1 and 2; M (black ellipse), the conserved WDXXEPXTY motif; Δ, truncated or substitution mutants; WALP, artificial TM sequence GWWLALALALALALALWWA. Substitutions of the conserved residues of TMH1 or TMH2 of TbMCU are indicated (multiple substitutions were generated, because single substitutions did not significantly alter protein-protein interaction). (B) Growth assay of the yeast NMY51 strain expressing the bait (TbMCU or TbMCUΔ1 to -Δ8) together with the prey (TbMCUc or TbMCUd) on SD selection agar plates as described for Fig. 5A. (C) A quantitative β-Gal activity assay of strain NMY51 coexpressing the bait-prey pairs as described for panel B determines their interaction strength. Each column represents the mean ± standard deviation (*n* = 3; 8 colonies for each independent experiment). (D and E) Expression level of each bait or prey as determined by immunoblot analysis using antitag antibodies, VP16 for the bait and HA for the prey, and hexokinase (HK) antibodies used as a loading control. Lanes M, molecular markers. (F) The scheme depicts wild-type TbMCUb, TbMCUc, TbMCUd, and their truncated or substitution mutant constructs as described for panel A. (G) Growth assay of the yeast strain NMY51 expressing the mutated bait (TbMCUΔ3, TbMCUΔ6-Δ8, TbMCUbΔ2-Δ4 TbMCUcΔ2-Δ4, or TbMCUdΔ2-Δ4) together with the mutated prey (TbMCUΔ3, TbMCUΔ6-Δ8, TbMCUbΔ2-Δ4 TbMCUcΔ2-Δ4, or TbMCUdΔ2-Δ4) on SD selection agar plates as in Fig. 5A. (H) Quantitative β-Gal activity assay of the strain NMY51 coexpressing the TbMCUC mutant bait-prey pairs to determine their interaction strength. Each column represents a mean ± standard deviation (*n* = 3; 8 colonies were used in each independent experiment). (I and J) Immunofluorescence validation of colocalization of the bait-prey interaction pairs, as in panels G and H, to the yeast plasma membrane. The PCC of the bait-prey pairs TbMCUΔ3-TbMCUcΔ1, TbMCUΔ3-TbMCUdΔ1, TbMCUcΔ1-TbMCUcΔ1, TbMCUdΔ1-TbMCUdΔ1, TbMCUbΔ1-TbMCUcΔ1, and TbMCUbΔ1-TbMCUdΔ1 are 0.6061, 0.5803, 0.6104, 0.6044, 0.6267, and 0.5266, respectively. Left images were obtained by DIC analysis. Scale bars = 5 µm. The merged images indicate colocalization (in yellow). (K and L) Validation by coimmunoprecipitation of interactions between the bait, i.e., TbMCUΔ3, TbMCUbΔ1, and TbMCUcΔ1 or TbMCUdΔ1, and the prey, i.e., TbMCUcΔ1 or TbMCUdΔ1, as in Fig. 5D and E.

To identify specific interacting domains or motifs that mediate the interactions between TbMCUC subunits, truncated or substitution TbMCU mutants (Fig. 6A) were generated and expressed as baits for MYTH assays (Fig. 6B and C). Deletion of the N- and/or C-terminal regions of TbMCU, designated TbMCUΔ1, TbMCUΔ2, and TbMCUΔ3 (Fig. 6A and Fig. S5B), did not affect the interaction with the subunit TbMCUc or TbMCUd (Fig. 6B and C), suggesting that the regions flanking the transmembrane helices (TMHs) of TbMCU are not involved in the protein-protein interactions. In contrast, TbMCU mutations of the conserved residues in TMH1 (Q213A, V216F, I217F, and F222A) or in TMH2 (Y235A, F236A, T241E, and Y248A) (Fig. S5C), named TbMCUΔ4 and TbMCUΔ5 (Fig. 6A), reduced the interaction with TbMCUc or TbMCUd (Fig. 6B and C), indicating that these residues are important for the TbMCU association with TbMCUc or TbMCUd. To confirm the critical role of the TMHs of TbMCU in the protein-protein interaction, TMH1, TMH2, or both TMHs of TbMCU were replaced with an artificial transmembrane “WALP” (19 amino acid residue long peptide) helix (GWWLALALALALALALWWA) (30) to generate the mutants TbMCUΔ6, TbMCUΔ7, and TbMCUΔ8 (Fig. 6A). The topology of these mutants did not change, as predicted with Protter (data not shown). Strikingly, the substitution of two TMHs did not alter their plasma membrane localization in yeast (Fig. S6B) but significantly disrupted their interaction with TbMCUc or TbMCUd (Fig. 6B and C). These results suggest that the TMHs of TbMCU are essential for the protein-protein interactions. Expression of the mutant proteins in yeast lysates was confirmed by Western blot analyses using α-VP16 and α-HA antibodies to detect the baits and preys, respectively (Fig. 6D and E).

10.1128/mBio.01700-18.6 FIG S6 Immunofluorescence localization of truncated and substitution mutants to the yeast plasma membrane. (A) Expression level of each of the baits or preys determined by immunoblotting using antitag antibodies, i.e., VP16 for the bait, HA for the prey, and hexokinase for the loading control. (B) TbMCUΔ1-, TbMCUΔ2-, TbMCUΔ3-, TbMCUΔ4-, TbMCUΔ5-, TbMCUΔ6-, TbMCUΔ7-, and TbMCUΔ8-transformed NMY51 cells were stained with anti-VP16 (1:100) and analyzed by immunofluorescence microscopy. Scale bars = 5 µm. Left images were obtained by DIC microscopy. (C to E) TbMCUbΔ2-, TbMCUbΔ3-, TbMCUbΔ4-, TbMCUcΔ2-, TbMCUcΔ3-, TbMCUcΔ4-, TbMCUdΔ2-, TbMCUdΔ3-, and TbMCUdΔ4-transformed NMY51 cells were stained with anti-VP16 (1:100) and analyzed by immunofluorescence microscopy. Scale bar, 5 µm. Left images were obtained by DIC microscopy. Download FIG S6, JPG file, 1.1 MB. Copyright © 2018 Huang and Docampo. 2018 Huang and Docampo This is an open-access article distributed under the terms of the Creative Commons Attribution 4.0 International license.

To determine whether the regions flanking the TMHs of TbMCUb, TbMCUc, and TbMCUd are involved in the protein-protein interactions, we generated the truncated mutations TbMCUbΔ1, TbMCUcΔ1, and TbMCUdΔ1 (Fig. 6F and Fig. S5B), which, like TbMCUΔ3, contained only the two conserved TMHs and pore regions of these proteins. MYTH assays revealed that the mutations, like that of TbMCUΔ3, did not affect the protein-protein interactions (Fig. 6G and H). The interactions among these TbMCUC truncation mutations were confirmed by coimmunoprecipitation (Fig. 6K and L) and immunofluorescence subcellular colocalization (Fig. 6I and F). On the other hand, to validate the important role of TMHs in the interactions, we replaced the TMH1 and/or TMH2 of TbMCUb, TbMCUc, and TbMCUd with the artificial transmembrane WALP helices to generate the mutations TbMCUbΔ2/Δ3/Δ4, TbMCUcΔ2/Δ3/Δ4, and TbMCUdΔ2/Δ3/Δ4 (Fig. 6F). Interestingly, the replacement of the TMHs of TbMCUb, TbMCUc, or TbMCUd by the WALP helices significantly disrupted the interactions (Fig. 6G and H), while the expression and localization of these proteins to the yeast plasma membrane were not altered (Fig. S6C to E). Like TbMCUΔ8 (Fig. 6B and C), the replacement of both TMHs of these proteins (TbMCUb4, TbMCUcΔ4, and TbMCUdΔ4) with the WALP helices abolished the protein-protein interactions (Fig. 6G and H). These studies therefore suggest that the TMH-TMH interactions of TbMCUC subunits, particularly the TMH2-TMH2 interaction, play a pivotal role in the oligomerization of TbMCUC subunits that form a hetero-hexameric MCU complex *in vivo.* Expression of the mutant proteins in yeast lysates was confirmed by Western blot analyses using α-VP16 and α-HA antibodies to detect the baits and preys, respectively (Fig. S6A).

## DISCUSSION

This study has discovered two novel pore-forming subunits (TbMCUc and TbMCUd) of the MCUC of T. brucei that are essential for mitochondrial Ca^2+^ uptake. We also defined their direct physical interactions with other subunits (TbMCU and TbMCUb), which suggest the formation of a hetero-hexameric MCU complex.

These two new paralogs of MCU are absent in the mammalian host, and examination of GenBank databases found them only in trypanosomatids. Both TbMCUc and TbMCUd have mitochondrial targeting signals, two transmembrane domains, and a modified Ca^2+^ selectivity filter (conserved WDXXEPXTY motif). Downregulation of their expression by RNAi significantly decreased while their overexpression significantly increased mitochondrial Ca^2+^ uptake without affecting cell growth in rich media or the mitochondrial membrane potential.

In agreement with results reported for T. cruzi (28), but in contrast to results described for HeLa cells (16), the T. brucei ortholog of mammalian MCUb (TbMCUb) did not have a dominant negative effect on mitochondrial Ca^2+^ uptake when overexpressed, and it reduced instead of increased mitochondrial Ca^2+^ uptake when its expression was downregulated. TbMCUb does not have a typical MTS, although it has a possible cleavage site for a signal sequence between amino acids 51 and 52, and our *in situ* tagging and overexpression studies localized it to the mitochondrion. The lack of a typical MTS also occurs in other subunits of the MCU complex in other trypanosomatids (Fig. S1A). Protein expression of TbMCUb could only weakly be downregulated by RNAi in BSF trypanosomes, explaining its weak inhibitory effect on mitochondrial Ca^2+^ uptake in the mutants. As happens with TbMCUc and TbMCUd, downregulation or overexpression of TbMCUb did not affect growth in rich media or the mitochondrial membrane potential. However, growth was significantly affected when TbMCUc and TbMCUd mutants were grown in glucose-deficient media, in agreement with the relevance of mitochondrial metabolism in PCF trypanosomes under low-glucose conditions (31).

All four subunits involved in mitochondrial Ca^2+^ uptake, TbMCUb, TbMCUc, TbMCUd, and TbMCU, colocalize with MitoTracker to the mitochondria of PCF and BSF trypanosomes. They interact with each other, as revealed by the formation of large protein complexes detected by blue native gel separation of mitochondrial proteins and labeling with antibodies against all the subunits and by coimmunoprecipitation of the subunits. To confirm these interactions, we used the MYTH technology. Although this technique detects mostly binary interactions between integral membrane proteins, by transforming yeast with genes encoding two subunits at a time, we were able to demonstrate the strength of their interactions, providing proof of concept for a new application of this technique. There were strong interactions of TbMCU with TbMCUc and TbMCUd, of TbMCUb with TbMCUc and TbMCUd, and of TbMCUs and TbMCUd with themselves, and there were weak interactions of TbMCU with T. cruzi MCUb (TcMCUb) or of TbMCUc with TbMCUd. The results suggest the formation of a hetero-hexameric complex, as shown in Fig. S4B. Our approach could be adopted to study other oligomeric complexes. It is interesting to note that previous studies with recombinant MCU from Caenorhabditis elegans showed that the purified protein with its amino-terminal domain (NTD) deleted formed homo-pentamers *in vitro* (19).

We could also obtain evidence that TM helix 1 (TMH1) and especially 2 (TMH2) of each of the four subunits of the complex are important for their interaction. Deletions of the C- and N-terminal regions of the proteins did not affect their membrane localization or their protein-protein interactions, while mutations in TbMCU TMH1 and TMH2 or their replacement with an artificial WALP helix (30) in any of the subunits greatly decreased their interactions without affecting their membrane localization. It is interesting to note that the N-terminal domain of the C. elegans MCU was shown to be nonessential for Ca^2+^ uptake but that TMH2 forms the inner core of the Ca^2+^ channel (19), suggesting a dual role for this helix. Only TbMCU and TbMCUd contain coiled-coil motifs. These are ubiquitous protein domains that mediate specific homo- and heteromeric protein-protein interactions among a wide range of proteins (32). However, split-ubiquitin MYTH assays with mutants lacking these domains showed that they are not relevant for interactions between the MCU complex subunits.

MCU paralogs are weakly expressed in trypanosomes, and endogenous tagging with high-performance probes was necessary for localization and protein-protein interaction studies. These tags consist of green fluorescent protein scaffolds containing numerous copies of peptide epitopes that simultaneously bind IgG antibodies at each location (spaghetti monsters) (25–27). This method allowed the use of cells with three of the subunits tagged with different epitopes and will be useful for localization and coimmunoprecipitation studies with other oligomeric and weakly expressed proteins in trypanosomes.

In summary, we detected two novel subunits of the T. brucei MCU complex, TbMCUc and TbMCUd. The results indicate that these proteins, as well as TbMCU and TbMCUb, are essential for mitochondrial Ca^2+^ uptake and suggest that they form a hetero-hexameric complex. Identification of the new components of the TbMCU complex may not only help to fully elucidate its structure but also provide new insights into understanding their evolutionary diversity.

## MATERIALS AND METHODS

### Cell culture.

T. brucei PCF trypanosomes (wild-type and 29-13 strains) and BSF trypanosomes (single-marker [SM] strain) were used. PCF 29-13 trypanosomes (*T7 RNAP NEO TETR HYG*) coexpressing T7 RNA polymerase and the *Tet* repressor were a gift from George A. M. Cross (Rockefeller University, NY) (33) and were grown in SDM-79 medium (34) supplemented with hemin (7.5 µg/ml) and 10% heat-inactivated fetal bovine serum (FBS) and were also grown at 27°C in the presence of G418 (15 µg/ml) and hygromycin (50 µg/ml) to maintain the integrated genes for T7 RNA polymerase and the tetracycline repressor, respectively. BSF trypanosomes (single-marker strain) were also a gift from G. A. M. Cross (33) and were grown at 37°C in HMI-9 medium (35) supplemented with 10% FBS, 10% serum plus (JRH Biosciences, Inc.), and 2.5 µg/ml G418.

### Ca^2+^ uptake by digitonin-permeabilized T. brucei.

The uptake of Ca^2+^ by permeabilized T. brucei was assayed by fluorescence measurements at 28°C using calcium green-5N (7). For PCF, trypanosome cells were collected by centrifugation at 1,000 *× g* for 7 min and washed twice with cold buffer A with glucose (BAG) (Text S1). PCF cells were resuspended to a final density of 1 × 10^9^ cells per ml in BAG and kept on ice. For BSF, trypanosome cells were collected by centrifugation at 1,000 *× g* for 7 min and washed twice with cold separation buffer, which contained 44 mM NaCl, 55 mM d-glucose, 57 mM Na_2_HPO_4_, and 3 mM KH_2_PO_4_ at pH 8.0. BSF trypanosomes were resuspended to a density of 2 × 10^7^ cells per ml in the separation buffer and kept on ice. Before each experiment, a 10-ml aliquot of the BSF cells was concentrated to 50 μl by centrifugation at 1,600 *× g* at room temperature for 3 min. A 50-μl aliquot of PCF (5 × 10^7^ cells) or BSF (2 × 10^8^ cells) trypanosomes of the cell suspension was added to the standard reaction buffer (125 mM sucrose, 65 mM KCl, 10 mM HEPES–KOH buffer, pH 7.2, 1 mM MgCl_2_, 2.5 mM potassium phosphate; 2.45 ml) containing 1 μM calcium green-5N and the reagents indicated in the figure legends. Ca^2+^ uptake by the cells was initiated by the addition of 50 μM digitonin (for PCF trypanosomes) or 40 μM digitonin (for BSF trypanosomes). Fluorescence changes were monitored in an F-7000 fluorescence spectrophotometer (Hitachi), with excitation at 490 nm and emission at 525 nm. Permeabilized cells grown in the presence of tetracycline under the assay conditions used did not show any fluorescence (excitation at 390 nm and emission at 400 to 650 nm) that could be attributed to tetracycline or its complexes.

10.1128/mBio.01700-18.10 TEXT S1 Supplemental methods. Download Text S1, DOCX file, 0.05 MB. Copyright © 2018 Huang and Docampo. 2018 Huang and Docampo This is an open-access article distributed under the terms of the Creative Commons Attribution 4.0 International license.

### BN-PAGE and immunodetection.

The frozen T. brucei mitochondrial vesicles were thawed on ice and washed three times with 1× sucrose-Tris-EDTA (STE) buffer (Text S1) by centrifugation at 16,000 × *g* for 10 min at 4°C. The pellet was lysed in 1× STE buffer containing 750 mM amino-*n*-caproic acid (ACA) and 2% dodecylmaltoside (DDM) for 1 h on ice and cleared by centrifugation at 16,000 × *g* for 30 min at 4°C. The protein concentration of the cleared lysate was determined by a Bradford assay (Bio-Rad). One hundred micrograms of lysate was mixed with 4× Native PAGE sample buffer (Invitrogen) and Native PAGE 5% G-250 sample additive (Invitrogen) to a final concentration of 0.1% and then loaded onto 4 to 16% Native PAGE Novex bis-Tris gels. Gel electrophoresis running buffers were prepared according to the manufacturer’s instructions for the Invitrogen Native PAGE Novex bis-Tris gel system. Full-strength Native PAGE running buffer was cooled to 4°C before use, and electrophoresis was performed at 4°C using XCell SureLock Mini-Cell (Invitrogen). Native MARK unstained protein standard (Invitrogen) was used to estimate molecular weight. Gels were run at 100 V for 1 h. Voltage was then increased to 250 V to run for approximately 90 min until the dye marker approached the gel front. When the dye front migrated through 1/3 of the gel and electrophoresis was paused, the dark-blue cathode buffer (containing 0.1% Coomassie blue G-250) was replaced with light-blue cathode buffer (containing 0.04% Coomassie blue G-250), according to the manufacturer’s instructions. After electrophoresis was complete, blue Native PAGE (BN-PAGE) gels were incubated with shaking in 2× NuPAGE transfer buffer for 5 min and then transferred to a 0.45-μm Immobilon-P polyvinylidene difluoride (PVDF) membrane (Millipore) in 1× NuPAGE transfer buffer (Invitrogen) containing 20% methanol and 0.04% SDS at 100 V for 3 h at 4°C for Western blot analysis using a Bio-Rad Transblot apparatus. After transfer, membranes were incubated in 8% acetic acid with shaking for 15 min to fix the proteins, treated with methanol to remove any Coomassie blue G-250 dye background for 3 min, and then washed with distilled water for 5 min. The membranes were blocked with 10% nonfat milk in phosphate-buffered saline (PBS) containing 0.5% Tween 20 (PBS-T), washed with PBS-T, and probed with anti-TbMCU, anti-FLAG, anti-HA, or anti-V5 antibodies as described below.

### Split-ubiquitin MYTH assays. (i) Yeast strains and media.

The Saccharomyces cerevisiae NMY51 MYTH reporter strain [*MAT***a** *his3*Δ*200 trp1-901 leu2-3,112 ade2* LYS2::(*lexA*op)_4_-HIS3 *ura3*::(*lexA*op)_8_-*lacZ ade2*::(*lexA*op)_8_-ADE2 GAL4] was obtained from Creative Biolabs (NY, USA). Yeast cells were grown using standard microbial techniques and media (29, 36). Medium designations are as follows. YPDA is 1% (wt/vol) yeast extract, 2% (wt/vol) peptone, 2% (wt/vol) dextrose plus 100 µM adenine medium. SD medium is synthetic defined dropout medium consisting of 0.67% (wt/vol) Difco yeast nitrogen base without amino acids, 2% (wt/vol) dextrose, 2% (wt/vol) agar, 0.7% sodium phosphate dibasic, 0.3% sodium phosphate monobasic, Sunrise amino acid/nucleotide dropout mix (e.g., a complete supplement medium [CSM]-Leu-Trp-His-Ade dropout complete supplement mixture lacking leucine, tryptophan, histidine, and adenine), supplemented with or without 2 mM 3-amino-1,2,4-trizole (3-AT), a histidine analog and competitive inhibitor of the *His3* gene product.

### (ii) MYTH bait and prey constructs.

The full-length or truncated cDNAs of the TbMCU, TbMCUb, TbMCUc, and TbMCUd genes without 5′ nucleotide sequences encoding the putative mitochondrial targeting signals (MTS) were amplified from T. brucei genomic DNA by PCR using the corresponding specific forward and reverse primers (see Table S1 in the supplemental material), which were introduced SfiI sites, digested with SfiI at 50°C overnight, and then cloned in frame into SfiI-digested MYTH assay bait (pBT3-SUC) and prey (pPR3N) expression vectors (29) to generate a set of MYTH constructs (Table S2) as described in the legend of Fig. 6A and F. The double-stranded sequences of the cloned cDNA inserts that express proteins of TbMCUC subunits C-terminally fused to Cub-LexA-VP16 in pBT3-SUC or N-terminally fused to NubG-HA in pPR3N were confirmed by sequencing as indicated above. The mutated amino acid residues or artificial WALP (GWWLALALALALALALWWA) sequence(s) was introduced into or replaced the TM domains of TbMCUC subunits in the MYTH bait or prey expression vectors (as described above) by fusion PCR (37) using a Phusion site-directed mutagenesis kit, according to the manufacturer’s instructions.

10.1128/mBio.01700-18.7 TABLE S1 Primers used in this study. Download Table S1, DOCX file, 0.04 MB. Copyright © 2018 Huang and Docampo. 2018 Huang and Docampo This is an open-access article distributed under the terms of the Creative Commons Attribution 4.0 International license.

10.1128/mBio.01700-18.8 TABLE S2 Plasmids constructed in this study. Download Table S2, DOCX file, 0.02 MB. Copyright © 2018 Huang and Docampo. 2018 Huang and Docampo This is an open-access article distributed under the terms of the Creative Commons Attribution 4.0 International license.

### (iii) MYTH assays of interaction between TbMCUC baits and preys.

The recombinant MYTH bait and prey plasmids (Table S2) harboring full-length, truncated, or mutated TbMCUC subunits with the empty vectors as negative controls were cotransformed into the yeast NMY51 strain by lithium acetate (LiOAc)-mediated transformation as described previously (38) and cultured successively on the dual, triple, and quadruple SD media (SD medium minus Leu and Trp, SD medium minus Leu, Trp, and His, and SD medium minus Leu, Trp, His, and Ade [shortened to SD-2DO, SD-3DO, and SD-4DO, respectively]). After incubation at 30°C for 3 to 4 days, colonies grown on the selective SD medium plates were further screened by cultivating them on SD-4DO–5-bromo-4-chloro-3-indolyl-β-d-galactopyranoside (X-Gal) medium, and β-galactosidase (β-Gal) activity was measured as described below to test the expression of the reporter gene *lacZ*. MYTH colonies were analyzed by Western blotting, immunofluorescence microscopy, and coimmunoprecipitation as described below.

### (iv) β-Gal activity assays.

To detect β-Gal expression by *lacZ* with X-Gal (39), MYTH colonies were grown onto SD-DO selection plates containing 2 mM 3-amino-1,2,4-triazole (AT), 0.8 mg/ml β-Gal (X-Gal), and 1× buffered (BU) salts (0.7% Na_2_HPO_4_, 0.3% NaH_2_PO_4_·H_2_O, pH 7.0) at 30°C. Blue staining was recorded for 1 to 2 days. To verify or quantify the interaction strengths of proteins, β-Gal activity for *lacZ* with the substrate of *o*-nitrophenyl-β-d-galactopyranoside (ONPG) was measured by a β-Gal microplate plate assay using a yeast β-Gal assay kit according to the manufacturer’s protocol. Briefly, single MYTH colonies were transferred with pipette tips to microcentrifuge tubes, each containing 150 µl SD liquid medium without Leu or Trp, mixed gently with a vortex mixer to create homogeneous solutions, and incubated with shaking at 30°C. After a 30-min incubation, 100 µl of each cell culture was added to a 96-well plate, and the optical density at 660 nm (OD_660_) of the solution was determined with a SpectraMax plate reader. Subsequently, 100 µl of fresh working solution containing a mixture of 1 volume of yeast protein extraction reagent (Y-per) with an equal volume of β-Gal assay buffer was added to each test well and mixed gently with a multichannel pipette. The reaction mixture was incubated at room temperature for approximately 30 min or until a color change was observed and then quenched by the addition of 1 M Na_2_CO_3_. The OD_420_ of each well was measured with the SpectraMax, and β-Gal units were calculated as follows: [units = 1,000 × OD_420_/(time × volume × OD_600_)]. One unit of β-Gal is defined as the amount that hydrolyzes 1 µmol of ONPG to *o*-nitrophenol and d-galactose per min per cell (40). The assay was repeated 3 times for a number of colonies (as indicated in the figures), followed by calculation of standard deviations. Statistical significance was calculated using Student’s *t* test.

### Statistical analyses.

All values are expressed as means ± standard deviations. Significant differences between treatments were compared using an unpaired Student *t* test. Differences were considered statistically significant at a *P* of *<*0.05, and *n* refers to the number of experiments performed. All statistical analyses were conducted using GraphPad Prism 5 (GraphPad Software, San Diego, CA).

10.1128/mBio.01700-18.9 TABLE S3 Antibodies used in this study. Download Table S3, DOCX file, 0.02 MB. Copyright © 2018 Huang and Docampo. 2018 Huang and Docampo This is an open-access article distributed under the terms of the Creative Commons Attribution 4.0 International license.
