# Supplementary figures and images for: The Mitochondrial Ca2+ Uniporter Complex (MCUC) of Trypanosoma brucei Is a Hetero-oligomer That Contains Novel Subunits Essential for Ca2+ Uptake
Source: mBio. 2018 Sep 18;9(5):e01700-18. doi: 10.1128/mBio.01700-18 (PMC6143741; doi:10.1128/mBio.01700-18)

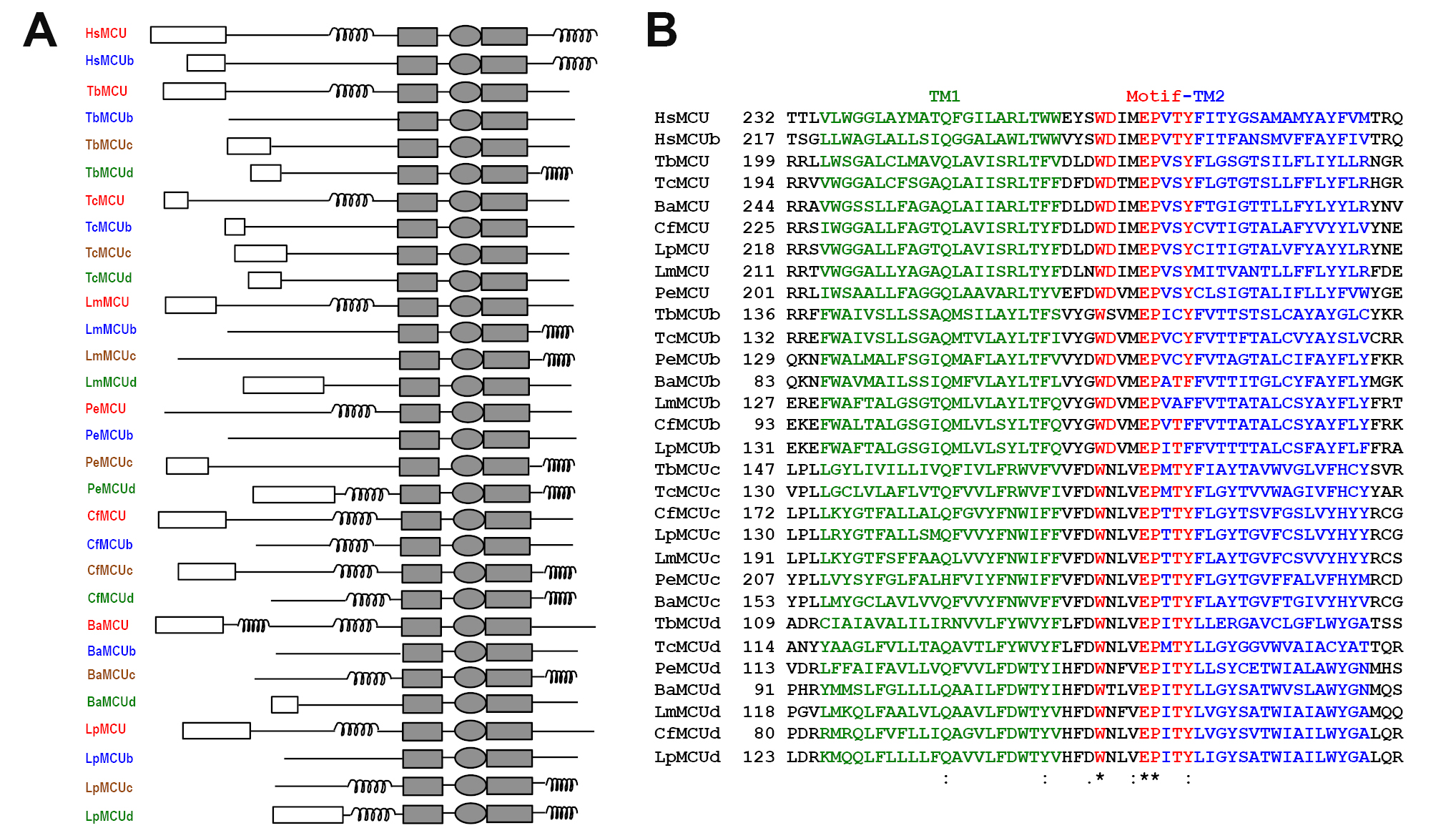

Supplement: FIG S1 [file mbo004184060sf1.jpg]

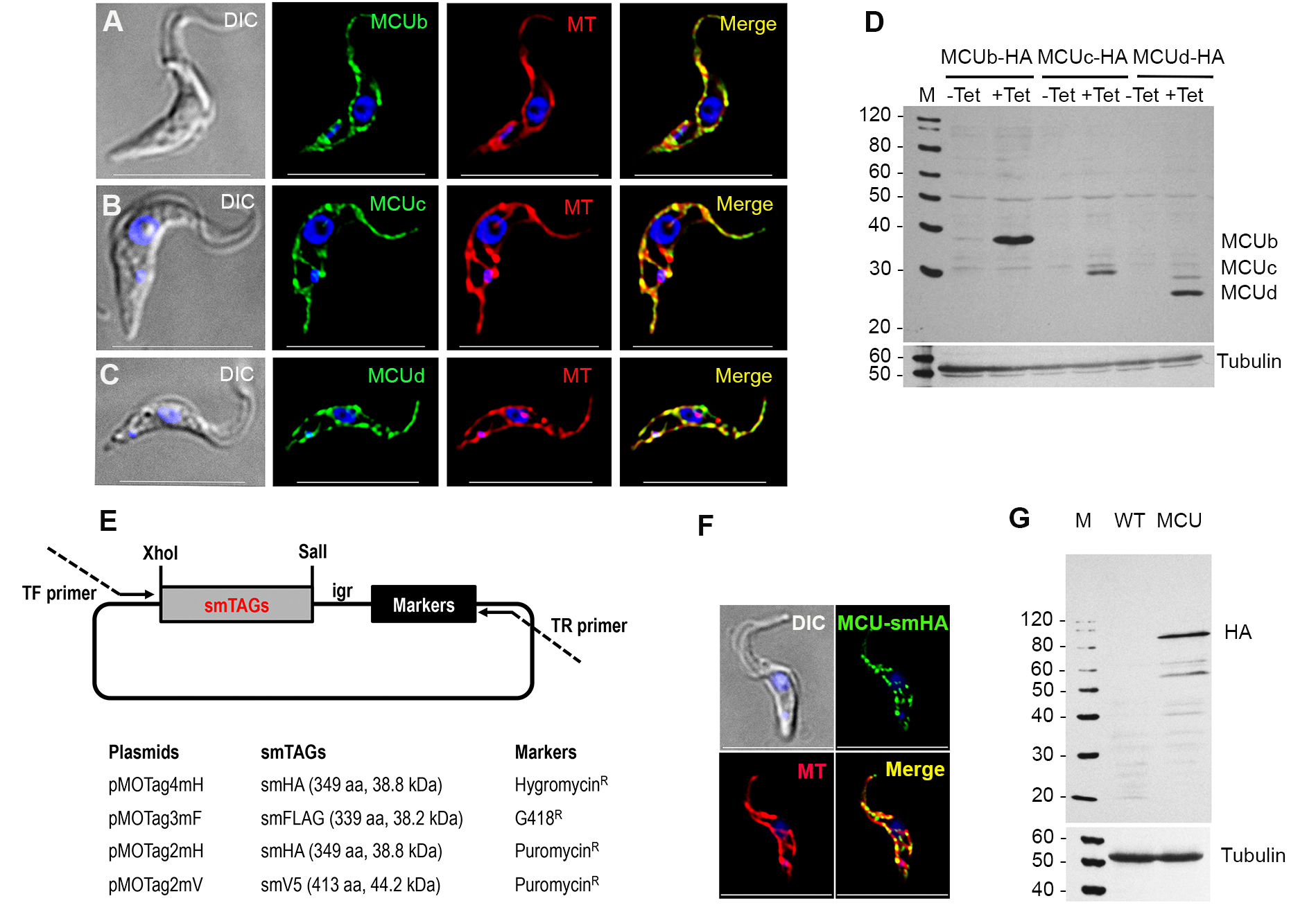

Supplement: FIG S2 [file mbo004184060sf2.jpg]

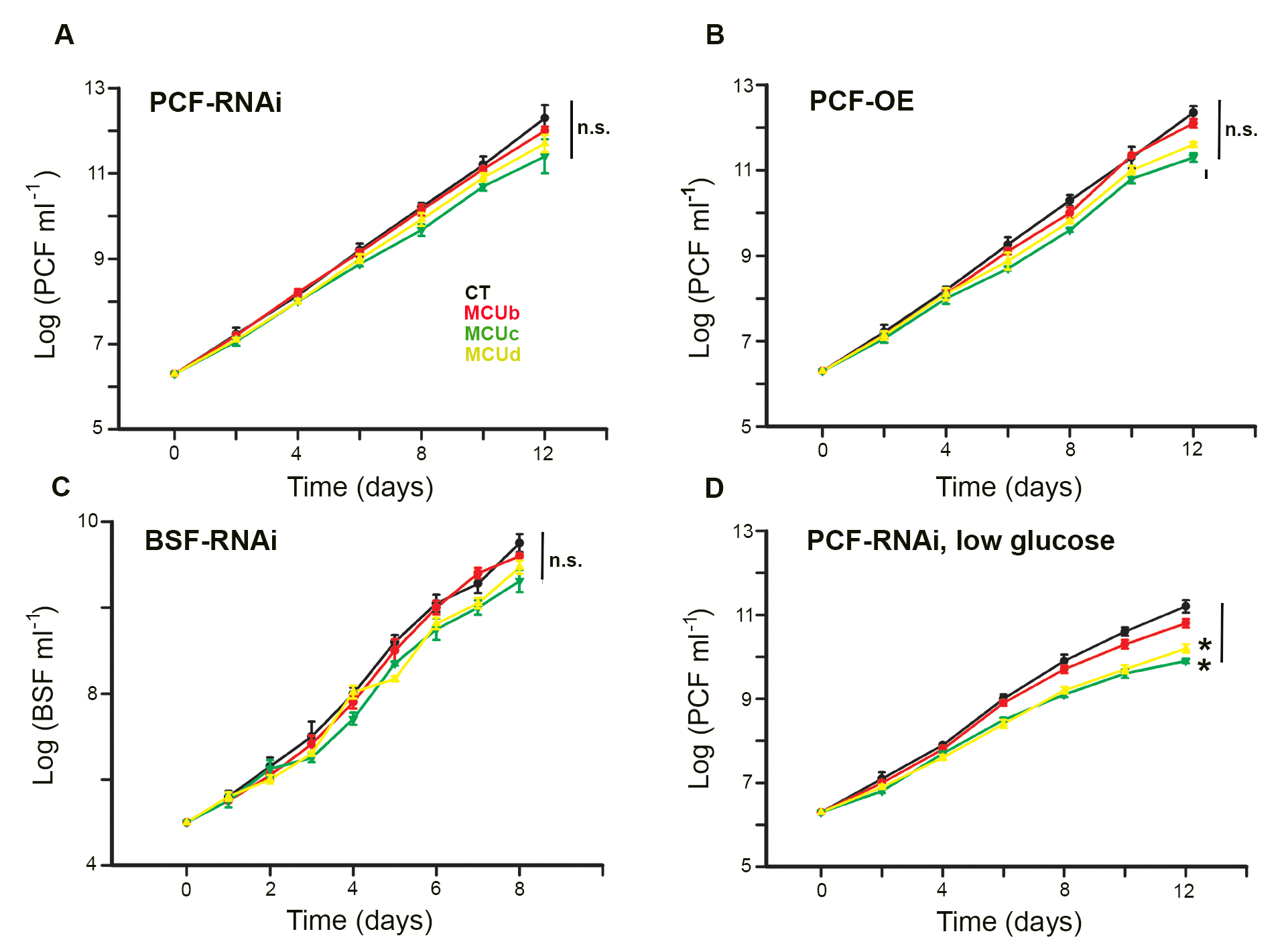

Supplement: FIG S3 [file mbo004184060sf3.jpg]

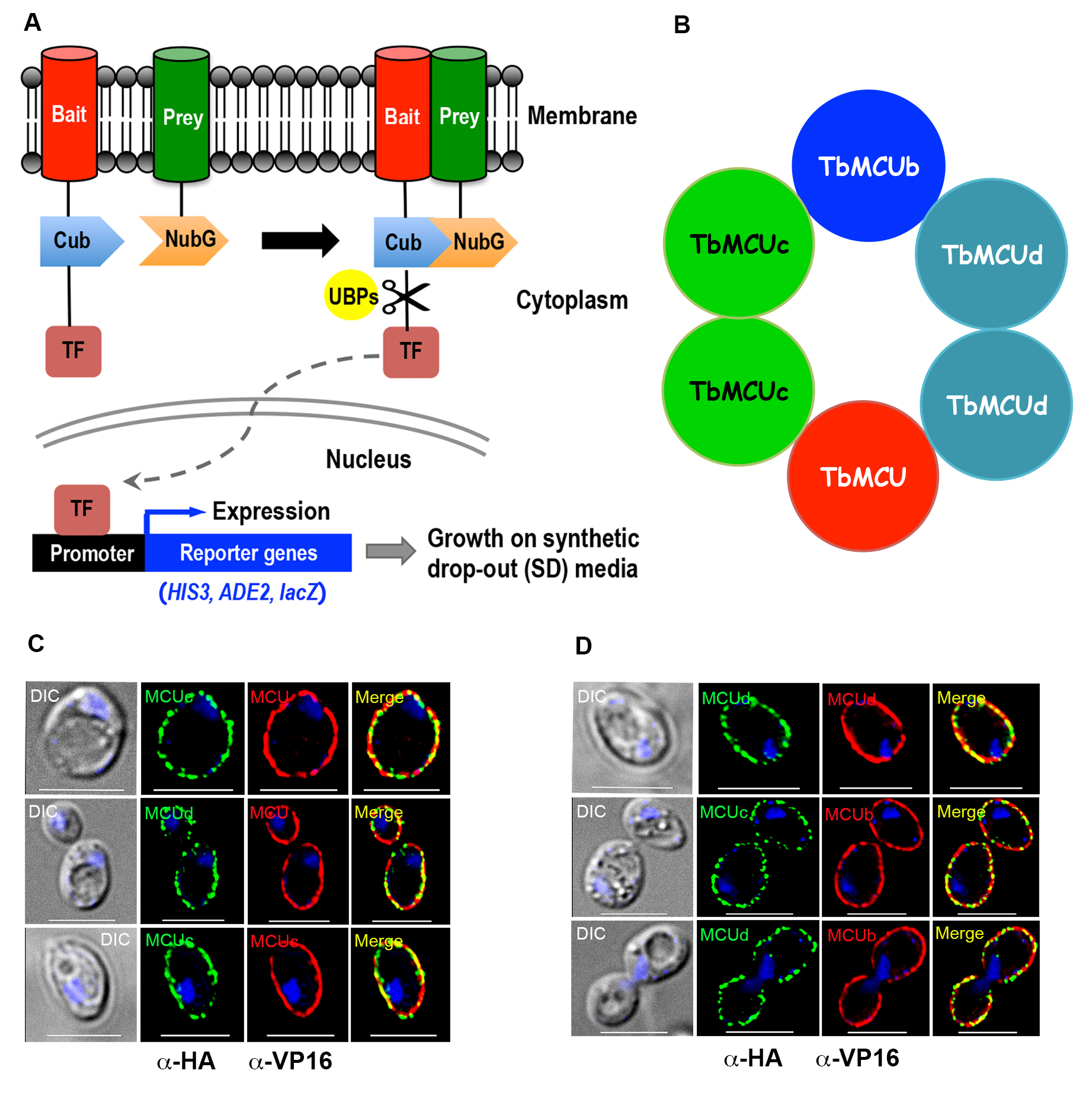

Supplement: FIG S4 [file mbo004184060sf4.jpg]

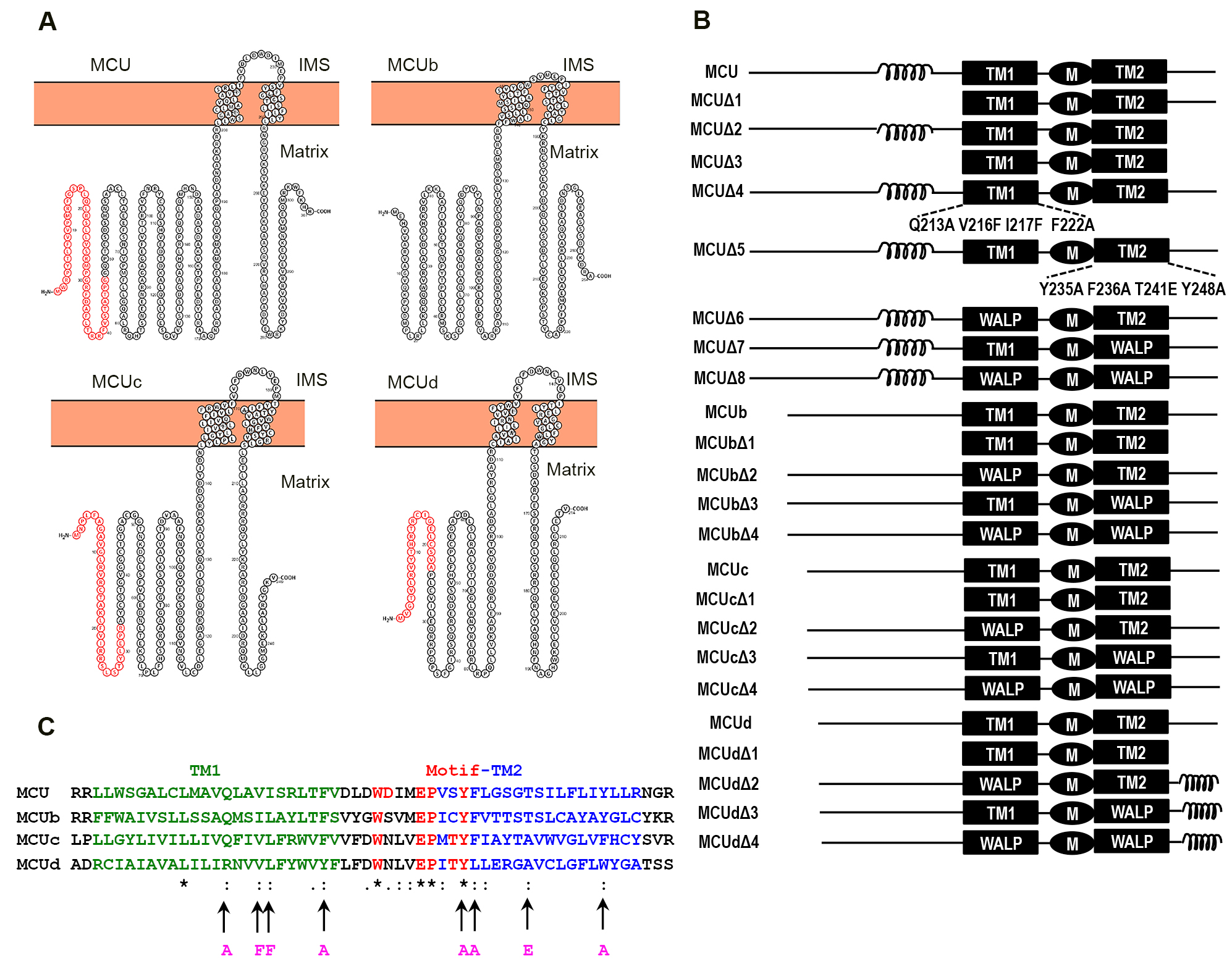

Supplement: FIG S5 [file mbo004184060sf5.jpg]

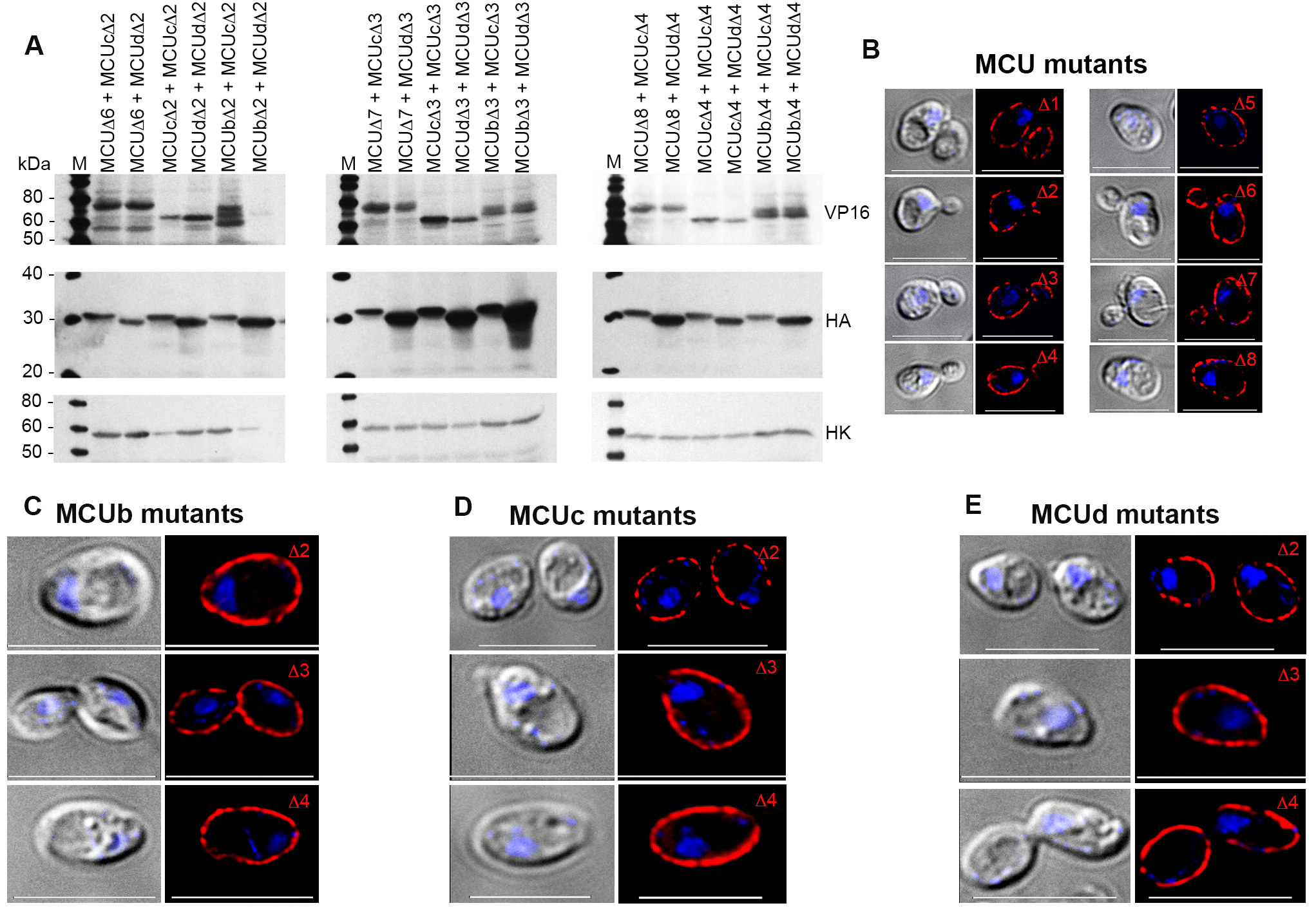

Supplement: FIG S6 [file mbo004184060sf6.jpg]
