# Supplementary material for: The Mitochondrial Ca2+ Uniporter Complex (MCUC) of Trypanosoma brucei Is a Hetero-oligomer That Contains Novel Subunits Essential for Ca2+ Uptake
Source: mBio. 2018 Sep 18;9(5):e01700-18. doi: 10.1128/mBio.01700-18 (PMC6143741; doi:10.1128/mBio.01700-18)
Supplement: TEXT S1 [file mbo004184060s1.docx]

**Supplementary Methods**

**Chemicals and reagents.** TRIzol reagent, MagicMark XP protein standards, Mito-Tracker Red CMXRos, Calcium Green-5N, Alexa-conjugated secondary antibodies, NativePAGE Novex BIS-Tris Gel System (4-16%), Bis-Tris Gels, Sample Buffer, Running Buffer, 5% G-250 Sample Additive, Transfer Buffer, NativeMark Unstained Protein Standard, ultra X-β-Gal, Yeast β-galactosidase Assay Kit, Pierce BCA Protein Assay Kit, mouse monoclonal antibody against FLAG, and Phusion High-Fidelity DNA polymerases, Phusion Site-Directed Mutagenesis Kit, Enhanced chemiluminescence (ECL) detection kit, Pierce Protein A/G Plus Agarose, Pierce Classic Protein G IP kit, Pierce HA Tag IP/Co-IP kit, and Pierce ECL Western blotting substrate were purchased from Thermo Fisher Scientific Inc. (Rockford, IL). Difco yeast nitrogen base w/o amino acids, In-Fusion HD Cloning kit and Advantage RT-for-PCR kit were purchased from Clontech (Mountain View, CA). Restriction endonucleases were purchased from New England Biolabs (Ipswich, MA). Complete, EDTA-free protease inhibitor cocktail tablets were purchased from Roche Applied Science (Indianapolis, IN). X-gal was purchased from Promega (Madison, WI). The pMOTag4H and pMOTag33M vectors were a gift from Dr. Thomas Seebeck (University of Bern, Bern, Switzerland) (1). The pMOTag2T and pLEW100v5b1d-BSD plasmids were a gift from Dr. George A. M. Cross (Rockefeller University, NY). The p2T7^Ti^ vector was a gift from Dr. John Donelson (University of Iowa, Iowa City, IA) (2). The pCAG_smFP FLAG, pCAG_smFP HA and pCAG_smFP V5 plasmids were a gift from Dr. Loren I. Looger (HHMI, Ashburn, VA) (3). The pBT3-SUC and pPR3N plasmids and yeast NMY51 MYTH reporter strain (4) were obtained from Creative Biolabs (NY, USA). The Bradford protein assay reagent, nitrocellulose membranes were from Bio-Rad (Hercules, CA). AMAXA Human T-cell Nucleofector kit was purchased from Lonza (Koln, Germany). Immobilon-P PVDF membrane was from Millipore (Billerica, MA). QIAquick gel extraction kit and MinElute PCR purification kit, and Protein G Agarose Resins were from Qiagen (Valencia, CA). CSM-Leu, CSM-Trp, CSM-Trp-Leu, CSM-Leu-Trp-His, CSM-Leu-Trp-His-Ade, yeast nitrogen base without amino acids and yeast culture grade agar were purchased from Sunrise Science Products (San Diego, CA). The primers were purchased from Integrated DNA Technologies (Coralville, IA). Mouse monoclonal antibody against HA (purified HA.11 clone 16B12) was purchased from Covance Inc. (Princeton, NJ). Rabbit polyclonal antibody against HA was purchased from Abcam (Cambridge, MA). Rabbit polyclonal antibodies against hexokinase were purchased from Rockland Antibodies and Assays (Limerick, PA). Rabbit polyclonal antibody against TbCyt c1 was a gift from Dr. Steve Hajduk (University of Georgia, GA). Rabbit polyclonal antibody against TbVDAC was a gift from Dr. Minu Chaudhuri (Meharry Medical College, TN). Carboxyatractyloside (CAT), oligomycin, Safranine O, carbonyl cyanide 4-(trifluoromethoxy)phenylhydrazone (FCCP), rabbit polyclonal antibody against VP16, rabbit polyclonal antibody against V5, anti-V5 agarose affinity gel, rabbit polyclonal antibody against tubulin, and all other reagents of analytical grade were from Sigma (St. Louis, MO). Production of anti-TbMCU antibodies was described before (5).

**Bioinformatics.** *T. brucei* MCUC subunits (TbMCU, TbMCUb, TbMCUc and TbMCUd) and human MCU homologs (HsMCU and HsMCUb) were used to identify the trypanosomatid homologues from TriTrypDB (<http://tritrypdb.org/tritrypdb/>) and GenBank (<https://www.ncbi.nlm.nih.gov/genbank/>) through BLASTP (iterative PSI-BLASPP) search. Proteins were annotated for mitochondrial targeting sequences (MTS) with Mitoprot (6) and TargetP (7). Transmembrane domains and coiled–coil (CC) domains were identified with TMHMM(8) and ExPASy Coils Server (<https://embnet.vital-it.ch/software/COILS_form.html>), respectively. Topology of membrane proteins was predicted with Protter (9). Amino acid substitutions were designed according to NCBI Amino Acid Explorer (<https://www.ncbi.nlm.nih.gov/>Class/Structure/aa/aa_explorer.cgi). Sequences from selective species were iteratively aligned with MUSCLE (<https://www.ebi.ac.uk/Tools/msa/muscle/>) and used to construct a phylogenetic tree with 500 bootstrap replicates using MEGA7 (10). The alignment and architecture of putative MCU domains were built by the bioinformatics analyses as described above.

**Generation of “Spaghetti monster” fluorescent proteins (smFPs) tagging constructs or cassettes.** The plasmids pMOTag4H, pMOTag33M(1) and pMOTag2T were used as backbones to construct “Spaghetti monster” fluorescent proteins (smFPs) with epitope tags (FLAG, HA or V5) plasmids (3). The plasmids were digested with XhoI and SalI to remove the sequences encoding 3xHA, 3xc-Myc or 3xTy1 epitope tags. smFLAG (1052bp), smHA (1082) and smV5 (1275bp) were PCR amplified from pCAG_smFP FLAG, pCAG_smFP HA and pCAG_smFP V5, respectively, using the primers smFP-F and smFP-R listed in Table S1 and cloned between the XhoI and SalI sites of the pMOTag vectors using the In-Fusion Cloning Kit (Clontech) to generate pMOTag4mH, pMOTag2mH, pMOTag3mF and pMOTag2mV listed in Table S2. The correct orientation and sequences of smFP-epitopes in the pMOTag vectors were confirmed by sequencing.

The one-step epitope-tagging protocol reported by Oberholzer et al. (1) was used to produce C-terminal smFP-epitope tagging cassettes of TbMCUb, TbMCUc, TbMCUd, (TriTrypDB gene ID numbers Tb427.10.300, Tb427tmp.02.1760, and Tb427.10.2150, respectively) for transfection of *T. brucei* PCF and BSF trypanosomes. In brief, the PCR forward and reverse primers included terminal 100-120 nucleotides of each ORF before its stop codon and the reverse complement of the first 100-120 nucleotides of the 3’UTR, respectively, followed in frame by the 21-26 nucleotides of the backbone sequences of pMOTag-smFP vector series (Table S2). The smFP-epitope tagging cassettes containing an antibiotic resistant gene as a selection marker (hygromycin, G418, or puromycin) were generated for cell transfection by PCR using pMOTag2mH, pMOTag4mH, pMOTag3mF, or pMOTag2mV (Fig. S2E, Table S2), as template with the corresponding PCR primers of the gene (Table S1).

**Isolation of *T. brucei* mitochondrial vesicles.** Crude mitochondrial vesicles were isolated from triple smFP-tagged TbMCUC PCF by hypotonic lysis as described(11) with some modifications. Briefly, mid-log phase PCF (~4x10^9^ cells in total) were harvested by centrifugation at 1,000 x *g* for 7 min at room temperature (RT) and washed twice with buffer A with glucose (BAG, 116 mM NaCl, 5.4 mM KCl, 0.8 mM MgSO_4_, 5.5 mM D-glucose and 50 mM Hepes at pH 7.0). The washed cells were resuspended in 3 ml DTE (1 mM Tris, 1 mM EDTA, pH 8.0), disrupted by 5 strokes in a Dounce homogenizer with tight-fitting pestle B, and passed through a 26-gauge needle at high pressure at 4^o^C. 60% sucrose was immediately added to a final concentration of 250 mM. After mixing, the lysate was centrifuged at 15,000 x *g* for 10 min at 4°C. The organelle-enriched pellet was re-suspended in 0.6 ml STM (250 mM sucrose, 20 mM Tris-HCl pH 8.0, 2 mM MgCl_2_), supplemented with a final concentration of 3 mM MgCl_2_ and 0.3 mM CaCl_2,_ and incubated with 9 μg/ml DNase I for 1 hr on ice. Then an equal volume of STE buffer (250 mM sucrose, 20 mM Tris-HCl pH 8.0, 2 mM EDTA pH 8.0) was added and the material was centrifuged at 15,000 x *g* as described above. The pellets enriched mitochondrial vesicles were washed twice with STE buffer and kept in 1 x STE buffer containing 50% glycerol at -80°C.

**RNAi constructs.** To knockdown the expression of the *TbMCUb*, *TbMCUc,* or *TbMCUd* genes by double-stranded RNA expression, the inducible T7 RNA polymerase-based protein expression system and the p2T7^Ti^ vector with dual-inducible T7 promoters were employed. cDNA fragments (ranging from 459 to 616bp) of the genes targeted to nucleotides (*TbMCUb*: 166-624, *TbMCUc*: 87-703*,* *TbMCUd*: 19-597) of the open reading frames (ORFs) were amplified using the forward and reverse primers listed in Table S1, digested with restriction enzymes (BamHI and HindIII), and cloned into p2T7^Ti^ vector. The recombinant constructs were confirmed by sequencing at the DNA Analysis Facility at Yale University (New Heaven, CT), NotI-linearized, and purified with QIAGEN’s DNA purification kit for cell transfections. *TbMCUb*, *TbMCUc*, and *TbMCUd* have novel nucleotide sequences without homologues (> 20 nucleotide identity) in *T.* *brucei* genome/transcript databases (TriTrypDB), suggesting the absence of any other potential gene targets.

**RT-PCR.** Total RNA was isolated with TRIzol reagent, treated with DNA-*free,* and reverse-transcribed using Advantage RT-for-PCR kit following the manufacturer’s instructions. Transcripts of *TbMCUb, TbMCUc* and *TbMCUd* were determined by reverse transcription (RT)-PCR. First-strand cDNAs from each sample were used in PCR reactions with the same set of gene-specific primers (Table S2) as RNAi constructs. Control RT-PCR reactions were conducted on each sample with the primers Tub-F and Tub-R (Table S2) designed from the constitutively expressed tubulin gene (TriTrypDB Tb427.01.2390). PCR products were analyzed on agarose gel and stained in ethidium bromide.

**HA-tagged TbMCUb, TbMCUc and TbMCUd.** The open reading frames (ORF) of TbMCUb (768 bp), TbMCUc (753 bp) or TbMCUd (648 bp) with 6 additional nucleotides prior to the start codons were amplified from *T. brucei* genomic DNA by PCR using the corresponding forward and reverse primers listed in Table S1, which introduced the restriction endonuclease SpeI or HindII and XbaI sites, respectively. The PCR products were digested with SpeI or HindIII and XbaI, and cloned in frame into the enzyme-cut pLEW100HA-BSD vector to generate pLEW100HA-BSD(*TbMCUb*), pLEW100100HA-BSD(*TbMCUc*) and pLEW100HA-BSD(*TbMCUd*), respectively (Table S2). The *T. brucei* expression vector pLEW100HA-BSD (Table S2) was created in three steps. First, pLEW100v5b1d-BSD was digested with HindIII and BamHI to remove the luciferase gene. Second, a DNA segment containing a multiple cloning site (including HindIII, SpeI, NdeI, HpaI and XbaI) and a cDNA encoding three repeats of the hemagglutinin (HA) peptide (YPYDVPDYA) followed by a stop codon and a BamHI site was purchased from GenScript (USA). Third, the synthetic cDNA flanking HindIII and BamHI sites was cloned into the enzyme-cut pLEW100v5b1d-BSD to generate pLEW100HA-BSD for C-terminal HA-epitope tagging. All of the relevant regions of the plasmids were confirmed by sequencing. The correct constructs pLEW100HA-BSD(*TbMCUb*)*,* pLEW100HA-BSD(*TbMCUc*) and pLEW100HA-BSD(*TbMCUd*) with inducible T7 RNA polymerase-based protein expression system were linearized by NotI and transfected into *T. brucei* PCF 29-13 trypanosomes.

**Cell transfections and growth assays.** Mid-log phase PCF trypanosomes (~5 × 10^6^ cells/ml) were harvested by centrifugation at 1,000 x *g* for 7 min, washed with Cytomix buffer (2 mM EGTA, 3 mM MgCl_2_, 120 mM KCl, 0.5% glucose, 0.15 mM CaCl_2_, 0.1 mg/ml BSA, 10 mM K_2_HPO_4_/KH_2_PO_4_, 1 mM hypoxanthine, 25 mM Hepes, pH 7.6) and re-suspended in 0.45 ml of the same buffer at a cell density of 2.5 × 10^7^ cells/ml. The washed cells were mixed with 50 µl of NotI-linearized plasmid DNA or purified PCR products (10 μg) in a 0.4-cm electroporation cuvette and subjected to two pulses from a Bio-Rad Gene Pulser electroporator set at 1.5 kV and 25 µF. The stable transformants were obtained in SDM-79 medium supplemented with 15% FBS plus appropriate antibiotics (15 μg/ml G418, 50 μg/ml hygromycin, 5 μg/ml phleomycin, 10 μg/ml blasticidin or/and 2 μg/ml puromycin).

For the BSF, 10 µg of NotI-linearized plasmid DNA (<10 µl) were used per 4 x 10^7^ mid-log phase cells in 100 µl AMAXA Human T-cell Nucleofector solution. Electroporation was performed using 2 mm gap cuvettes with program X-001 of the AMAXA Nucleofector. Following each transfection, stable transformants were selected and cloned by limiting dilution in HMI-9 medium containing 15% FBS with appropriate antibiotics (2.5 µg/ml G418, 5 μg/ml blasticidin, 2.5 µg/ml phleomycin or/and 0.1 μg/ml puromycin) in 24-well plates. Antibiotic-resistant clones were further characterized as described below. The correct smFP-epitope-tagging of the target genes was confirmed by PCR followed by sequencing and Western blot analyses. Overexpression or RNAi of *TbMCUb*, *TbMCUc* and *TbMCUd* was induced with 1 µg/ml fresh tetracycline when the cells were at a density of 2 x 10^6^ PCF or 1 x 10^5^ BSF/ml.

For cell growth assays, *TbMCUb*, *TbMCUc* and *TbMCUd* RNAi or overexpressing PCF trypanosomes were cultivated in SDM-79 or in a glucose-depleted medium (SDM-80) containing 5.2 mM L-proline (12) in the absence or presence of tetracycline. *TbMCUb*, *TbMCUc* and *TbMCUd* RNAi BSF trypanosomes were cultivated in HMI-9 in the absence or presence of tetracycline. The cells were counted using a hemocytometer and growth curves were generated for clones or cell lines over a period of 12 days for PCF and 8 days for BSF.

**Mitochondrial membrane potential and phosphorylation.** The mitochondrial membrane potential *in situ* was analyzed spectrofluorometrically by using safranine O as the probe (5, 13)*. T. brucei* PCF and BSF trypanosomes were incubated at 28°C in the standard reaction buffer with additions as described in the figure legends. Fluorescence changes were monitored on a Hitachi 7000 spectrofluorometer (excitation wavelength = 496 nm; emission wavelength = 586 nm).

**Immunofluorescence microscopy.** To determine the localization and expression of TbMCUb, TbMCUc and TbMCUd in *T. brucei*, trypanosome live cells were labeled for 30 min with Mitotracker Red CMXRos (Invitrogen) at 50 nM in trypanosome culture medium. BSF trypanosomes were washed in ice-cold PBS with 1% glucose and fixed with 1% paraformaldehyde in PBS at 4°C for 1 h. PCF trypanosomes were washed with PBS and then fixed with 4% paraformaldehyde in PBS at room temperature for 1 h. The fixed parasites were washed twice with PBS, allowed to adhere to poly-L-lysine-coated coverslips and permeabilized with 0.3% Triton X-100/PBS for 3 min for PCF or 0.1% Triton X-100/PBS for 5 min for BSF. After blocking with PBS containing 3% BSA, 1% fish gelatin, 50 mM NH_4_Cl and 5% goat serum for 1 h, trypanosomes were stained in 3% BSA/PBS with the purified HA.11 clone 16B12 monoclonal antibody against HA (1:50), rabbit polyclonal antibody against HA (1:1,000), rabbit polyclonal antibody against FLAG (1:1,000), mouse monoclonal antibody against V5 (1:100), rabbit monoclonal antibody against V5 (1:500), mouse polyclonal antibody against TbMCU (1:100) as described in Table S3, for 1 h. After thoroughly washing with PBS containing 3% BSA, cells were incubated with Alexa 488-conjugated goat anti-mouse or anti-rabbit antibodies and Alexa 546-conjugated goat anti-rabbit or anti-mouse antibodies at 1:1,000 (Table S3) for 1 h.

Immunofluorescence of yeast was performed as described (14) with some modifications. Briefly, mid- to late-log phase yeast cells were centrifuged at 700 x *g* for 5 min and fixed with 4% formaldehyde in SD/-Leu/-Trp on a shaker (200 rpm) at 30^o^C for 1 h. Cells were collected by centrifugation, washed once with 1 ml of fresh medium, and incubated with DET (100 mM DTT, 20 mM EDTA. 20 mM Tris-HCl pH8.0) at RT for 5 min. After collecting the cells by centrifugation, the cell pellet was suspended in 1 ml of 0.9 M sorbitol/PBS pH7.4, added 20 mg/ml zymolyase to a final concentration of 100 μg/ml, and then incubated on a shaker (200 rpm) for 30-60 min at 37^o^C until cell walls were digested. Spheroplasts were washed gently with 0.9 M sorbitol/PBS, allowed to adhere to poly-L-lysine-coated coverslips, and permeabilized with 1% Triton X-100/0.9 M sorbitol/PBS pH7.4 for 10 min at RT. After blocking with PEM (100 mM Pipes pH7.0, 1 mM EGTA, 0.1 mM MgSO_4_, 1% BSA and 0.1% NaN_3_) for 1 h, spheroplasts were labeled in PEM with the purified HA.11 clone 16B12 monoclonal antibody against HA (1:50) and rabbit polyclonal antibody against VP16 (1:100) as described in S3 Table, for 1 h. After thoroughly washing with PEM, cells were incubated with Alexa 488-conjugated goat anti-mouse antibody and/or Alexa 546-conjugated goat anti-rabbit antibody at 1:1,000 (Table S3) for 1 h.

After labeled with primary and secondary antibodies, the trypanosome or yeast cells on the coverslips were counterstained with 4′,6-diamidino-2-phenylindole (DAPI) before mounting with Gold ProLong Gold antifade reagent (Molecular Probes). Differential interference contrast and fluorescent optical images were captured using an Olympus IX-71 inverted fluorescence microscope with a Photometrix CoolSnap^HQ^ charge-coupled device camera driven by DeltaVision software (Applied Precision, Seattle, WA). Images were deconvolved for 15 cycles using Softwarx deconvolution software. Pearson’s correlation coefficients (PCC) were calculated using the Softwarx software by measuring the whole-cell images.

**Immunoprecipitation studies.** *T. brucei* MCU complex was immunoprecipitated from triple smFP-tagged TbMCUC PCF cell line under native conditions using antibodies against TbMCU, FLAG, HA or V5 with Pierce Classic Protein G IP kit or HA-Tag IP/Co-IP kit according to the manufacturer’s instructions. Briefly, cell pellets from ~1.2 x 10^10^ cells were washed once in PBS with 6 mM glucose and lysed for 30 min on ice with 1% Triton X-100 in 18 ml IPP150 (10 mM Tris-HCl, pH8.0, 150 mM NaCl, 0.1% NP40) containing complete protease inhibitors (Roche). The lysate was cleared of debris by centrifugation at 15,000 x *g* for 15 min at 4^o^C. The supernatant (cleared lysate) was collected and divided into five aliquots: one used for immunoprecipitation controls and the other used for experimental immunoprecipitations. For immunoprecipitation of the TbMCU complex, 10 µg of anti-TbMCU, anti-FLAG, anti-HA or anti-V5 antibodies diluted in PBS, pH 8.0, were bound to Pierce Protein A/G Plus Agarose on a mixer for 2 h at 4^o^C, washed twice with IPP150 and then incubated with aliquots of cleared lysate overnight with gentle rotation at 4^o^C. The agarose-bounded complexes were precipitated by centrifugation at 1,600 g for 5 min at 4^o^C, resuspended in 500 µl ice-cold IPP150 containing complete protease inhibitors, and then transferred to Pierce Spin Column. The protein-agarose mixtures were centrifuged at 6,000 g for 1 min, washed once in IPP150, and then washed three times in 0.5 ml of TBS-T (25 mM Tris-HCl, 0.15M NaCl, pH7.2, 0.05% Tween-20) by mixing and centrifugation. The bound protein complexes were eluted in 100 µl of Elution Buffer (pH 2.8) by centrifugation and immediately neutralized by adding 5 µl Tris, pH 9.5. Finally, 10 µl of each eluate (or immunoprecipitate) and 30 µl of cleared lysate were loaded on 10-12% SDS-PAGE gels for western blot analyses.

For yeast Co-IP analysis, MYTH colonies grown on SD-Leu-Trp-His-Ade plates were incubated in 3 ml of SD-Leu-Trp liquid media (for lysates), 10 ml of SD-Leu (bait expression) or SD-Trp (prey expression) liquid media (for IPs) with shaking at 230 rpm at 30^o^C overnight. The cells were harvested and washed once with distilled H_2_O. The cell pellets were suspended in 0.9 M sorbitol/PBS pH7.4, added 20 mg/ml zymolyase to a final concentration of 100 μg/ml and then incubated on a shaker (200 rpm) for 30-60 min at 37^o^C until cell walls were digested. For lysate preparations, spheroplasts were washed gently with 0.9 M sorbitol/PBS and lysed in RIPA buffer (150 mM NaCl, 20 mM Tris/HCl, pH7.5, 1 mM EDTA, 1% SDS, and 0.1% Triton X-100) containing complete protease inhibitors on ice for 1 h. The protein concentration was determined by using a Pierce BCA protein assay kit with the SpectraMax. For immunoprecipitation preparation, spheroplasts were washed gently with 0.9 M sorbitol/PBS and lysed on ice for 1 h with 1% Triton X-100 in IPP150 containing complete protease inhibitors. The lysate was cleared of debris by centrifugation at 15,000 g for 15 min at 4^o^C. Equivalents of cleared lysates from SD-Leu (bait) and SD-Trp (prey) liquid cultures were mixed and immunoprecipitated under native conditions using antibodies against HA and VP16 as described above.

**Western blot analyses*.*** The *T. brucei* cells were harvested and washed twice in PBS. The washed cells were lysed with RIPA buffer (150 mM NaCl, 20 mM Tris/HCl, pH 7.5, 1 mM EDTA, 1% SDS, and 0.1% Triton X-100) containing protease inhibitor tablet in ice for 1 h. The protein concentration was determined by using Pierce BCA protein assay kit with the microplate reader. The yeast NMY51 cells were digested with zymolyase and then lysed in RIPA buffer as described above.

Total cell lysates, mitochondrial vesicle lysates, or immunoprecipitation eluates were mixed with 2 × Laemmli sample buffer (BioRad) at 1:1 ratio (volume/volume), directly loaded (for *T. brucei* proteins) or loaded after boiling for 5 minutes (for yeast proteins). The separated proteins were transferred onto nitrocellulose membranes or Immobilon-P PVDF 0.45 μm membranes using a Bio-Rad transblot apparatus. The membranes were blocked with 10% non-fat milk in PBS-T at 4^o^C overnight. The blots were incubated with mouse antibodies against TbMCU (1:1,000), rabbit antibodies against FLAG (1:20,000), mouse antibodies against HA (1:1,000), rabbit antibodies against HA (1: 20,000), mouse antibodies against V5 (1:2,500), rabbit antibodies against TbVDAC (1:2,000), rabbit antibodies against VP16 (1:1,000), rabbit antibodies against TbCyt c_1_ (1:5,000), rabbit antibodies against hexokinase (1:20,000) or rabbit antibodies against tubulin (1:10,000) as described in S3 Table, for 1 h. After five washings with PBS-T, the blots were incubated with horseradish peroxidase conjugated anti-mouse or anti-rabbit IgG (H+L) antibody at a dilution of 1:15,000 (Table S3) for 1 h. After washing five times with PBS-T, the immunoblots were visualized using Pierce ECL Western blotting substrate according to the manufacturer’s instructions.

**REFERENCES**

1. Oberholzer M, Morand S, Kunz S, Seebeck T. 2006. A vector series for rapid PCR-mediated C-terminal in situ tagging of *Trypanosoma brucei* genes. Mol Biochem Parasitol 145:117-20.

2. LaCount DJ, Barrett B, Donelson JE. 2002. *Trypanosoma brucei* FLA1 is required for flagellum attachment and cytokinesis. J Biol Chem 277:17580-8.

3. Viswanathan S, Williams ME, Bloss EB, Stasevich TJ, Speer CM, Nern A, Pfeiffer BD, Hooks BM, Li WP, English BP, Tian T, Henry GL, Macklin JJ, Patel R, Gerfen CR, Zhuang X, Wang Y, Rubin GM, Looger LL. 2015. High-performance probes for light and electron microscopy. Nat Methods 12:568-76.

4. Lentze N, Auerbach D. 2008. Membrane-based yeast two-hybrid system to detect protein interactions. Curr Protoc Protein Sci Unit 19.17: 1-28. doi:10.1002/0471140864ps1917s52.

5. Huang G, Vercesi AE, Docampo R. 2013. Essential regulation of cell bioenergetics in Trypanosoma brucei by the mitochondrial calcium uniporter. Nat Commun 4:2865.

6. Claros MG, Vincens P. 1996. Computational method to predict mitochondrially imported proteins and their targeting sequences. Eur J Biochem 241:779-86.

7. Emanuelsson O, Brunak S, von Heijne G, Nielsen H. 2007. Locating proteins in the cell using TargetP, SignalP and related tools. Nat Protoc 2:953-71.

8. Krogh A, Larsson B, von Heijne G, Sonnhammer EL. 2001. Predicting transmembrane protein topology with a hidden Markov model: application to complete genomes. J Mol Biol 305:567-80.

9. Omasits U, Ahrens CH, Muller S, Wollscheid B. 2014. Protter: interactive protein feature visualization and integration with experimental proteomic data. Bioinformatics 30:884-6.

10. Kumar S, Stecher G, Tamura K. 2016. MEGA7: Molecular Evolutionary Genetics Analysis Version 7.0 for Bigger Datasets. Mol Biol Evol 33:1870-4.

11. Harris ME, Moore DR, Hajduk SL. 1990. Addition of uridines to edited RNAs in trypanosome mitochondria occurs independently of transcription. J Biol Chem 265:11368-76.

12. Lamour N, Riviere L, Coustou V, Coombs GH, Barrett MP, Bringaud F. 2005. Proline metabolism in procyclic *Trypanosoma brucei* is down-regulated in the presence of glucose. J Biol Chem 280:11902-10.

13. Vercesi AE, Bernardes CF, Hoffmann ME, Gadelha FR, Docampo R. 1991. Digitonin permeabilization does not affect mitochondrial function and allows the determination of the mitochondrial membrane potential of *Trypanosoma cruzi* in situ. J Biol Chem 266:14431-4.

14. van Suylekom D, van Donselaar E, Blanchetot C, Do Ngoc LN, Humbel BM, Boonstra J. 2007. Degradation of the hexose transporter Hxt5p in *Saccharomyces cerevisiae*. Biol Cell 99:13-23.
