# Supplementary material for: The Mitochondrial Ca2+ Uniporter Complex (MCUC) of Trypanosoma brucei Is a Hetero-oligomer That Contains Novel Subunits Essential for Ca2+ Uptake
Source: mBio. 2018 Sep 18;9(5):e01700-18. doi: 10.1128/mBio.01700-18 (PMC6143741; doi:10.1128/mBio.01700-18)
Supplement: TABLE S1 [file mbo004184060st1.docx]

**Table S1. Primers used in this study**

| **Name** | **Usage** | **Primer sequence (5’ to 3’)** | **Enzyme** |
| --- | --- | --- | --- |
| SmGFP-FF | Tagging | CGGGCCCCCCCTCGAGAGGAAAAAAGCGGCCGCCACC | *Xho*I |
| SmHA-FR | Tagging | GTCACACTTTGTCGACCGAACCGGTTTAAGCGTAGTCCGGGACATCG | *Sal*I |
| SmFLAG-FR | Tagging | GTCACACTTTGTCGACCGAACCGGTTTATTTATCATCGTCGTCTTTG | *Sal*I |
| SmV5-FR | Tagging | GTCACACTTTGTCGACCGAACCGGTTTAGGTACTATCCAGTC | *Sal*I |
| TbMCUb-TF | Tagging | CAGACACTTTGGTGGAAGGAAAGTCCCCTTCCACCGTGTGTGCACAACCATTTTTCATGGAAGCAGTGGAACTTTTGCGGGCCGTGCAAGCGGAAAGCGACAACAGTGGATTGGCTGAAGCTTCTTCTTCCGATACCAAAGACAGAGCTGGTACCGGGCCCCCCCTCGAG |  |
| TbMCUb-TR | Tagging | ATCATTCGAATAGTAGATGATGCAAGGTCTCTTGAGCTCTATCCAAATACGTATTACACATTCCAATATTATCGCTTTTCTTGCACTACTGCTACTTCCGCTAACCACTACGGAATCTTTATATTCTCCCCTTCATTGGTCTGCATTTGGCGGCCGCTCTAGAACTAGTGGAT |  |
| TbMCUc-TF | Tagging | AGACGCTACTAGCGGAGAGGCGGCGCCAGGTGTTATACAAGAGGGCTCGGATCGATGGGGCAGCCATTGATCGTCAGATGAAACTCCTTGGAGGAATGGAGAAACTACTTGCTCGCTACGGAAAGGTGGGTACCGGGCCCCCCCTCGAG |  |
| TbMCUc-TR | Tagging | AAGCACGAACACATGCACATCAACCCCGTGAAGCATCAGAATTAATTTAGATGAAAGAACGTTGTTTTTTGTTTTTAACGGGCGCCTACCGAAGAACTTCCCTGATCAACATCTCTTTTTTATGGCGGCCGCTCTAGAACTAGTGGAT |  |
| TbMCUd-TF | Tagging | CAATTCCTCGTGTCCCGTCGGACGCAACGGCTTTATGCGCAGCACAACTTTAACGCTGGACACTGGAAGGAGTTGGTGGTGGAGGTGGAGGGGCTTGAAGAGCAGCTGCGCGGGCTGGAGACTGTTGGTACCGGGCCCCCCCTCGAG |  |
| TbMCUd-TR | Tagging | AAGATTGATACATGAAGAAGCACACGATGAGCTTTTCTTTGTAACTTAAAGTTGTGCAACAGGCAGAATGCAAGAAGAAGATAGGACAGTCCACCTCCGAACCTTCAATGTGATGGAGGCTTGGCGGCCGCTCTAGAACTAGTGGAT |  |
| TbMCUb-IF | RNAi | CGGGATCCCCGTTGTGTGAGCGAATGTC | *Bam*HI |
| TbMCUb-IR | RNAi | CCCAAGCTTCAAAGTGTCTGAAGACGCCAAC | *Hin*dIII |
| TbMCUc-IF | RNAi | CGGGATCCCTTGGAACCACGTGCATACTG | *Bam*HI |
| TbMCUc-IR | RNAi | CCCAAGCTTGTTTCATCTGACGATCAATGGCTG | *Hin*dIII |
| TbMCUd-IF | RNAi | CGGGATCCCGCGTATATACACACCGCAC | *Bam*HI |
| TbMCUd-IR | RNAi | CCCAAGCTTCACCACCAACTCCTTCCAGTG | *Hin*dIII |
| TbMCUb-RF | RT-PCR | TCCAACCCGTACACACCGTTGTG |  |
| TbMCUb-RR | RT-PCR | TTAAGCTCTGTCTTTGGTATCGGAAG |  |
| TbMCUc-RF | RT-PCR | GCATACTGTAGCACTGGAGTCGG |  |
| TbMCUc-RR | RT-PCR | CTACACCTTTCCGTAGCGAGCAAG |  |
| TbMCUd-RF | RT-PCR | CCATTGTGCGTTATTTTGGAACAG |  |
| TbMCUd-RR | RT-PCR | TCAAACACTCTCCAGCCCGCGCAG |  |
| TbbTUB-F | RT-PCR | CGACTCTGTGCTCGATGTGTGC |  |
| TbbTUB-R | RT-PCR | CTGCAGCTTGCATCATGTTTTTCGC |  |
| TbMCUb-TG-F | OE | GGACTAGTAAAATACAATGGAGCACGTAGTAGCCAGGGTC | *Spe*I |
| TbMCUb-TG-R  TbMCUc-TG-F | OE  OE | GCTCTAGAAGCTCTGTCTTTGGTATCGGAAG  CCCAAGCTTAAAATCAAATGAACCCACTTTTTGCCGGCGCTG | *Xba*I  *Hin*dIII |
| TbMCUc-TG-R | OE | GCTCTAGACACCTTTCCGTAGCGAGCAAG | *Xba*I |
| TbMCUd-TG-F | OE | CCCAAGCTTAATCTTTCATGGTTGGAACAGTATTACG | *Hin*dIII |
| TbMCUd-TG-R | OE | GCTCTAGAAACACTCTCCAGCCCGCGCAG | *Xba*I |
| BT3-SUC-SF | MYTH | TGGCATGGATGTGCTCTG |  |
| BT3-SUC-SR | MYTH | GTAAGGTGGACTCCTTCT |  |
| BT3SUC-TbMCU-F | MYTH | ATTAACAAGGCCATTACGGCCGGGCAGCCGACTTGTAGTGACTC | *Sfi*I |
| BT3SUC-TbMCU-R | MYTH | AATGATTGGCCGAGGCGGCCCCGTGATGTTTAAACCACTTCCG | *SfiI* |
| BT3SUC-TbMCU-DF1 | MYTH | ATTAACAAGGCCATTACGGCCCTTCAACCCGCTATTGATAATGC | *Sfi*I |
| BT3SUC-TbMCU-DF2 | MYTH | ATTAACAAGGCCATTACGGCCCTCTTGTGGTCGGGTGCCCTCTG | *Sfi*I |
| BT3SUC-TbMCU-DR1 | MYTH | AATGATTGGCCGAGGCGGCCCCGCTCTTCACCCGCCCATTCCTG | *Sfi*I |
| BT3SUC-TbMCU-PM-F4 | MYTH | GCAGTCGCACTTGCATTTTTCTCACGGCTAACAGCTGTCGACCTTGAC |  |
| BT3SUC-TbMCU-PM-R4 | MYTH | GTCGACAGCTGTTAGCCGTGAGAAAAATGCAAGTGCGACTGCCATAAG |  |
| BT3SUC-TbMCU-PM-F5-1 | MYTH | GGGCAGTGGTGAATCGATCCTGTTTCTTATTGCTCTTCTCAGGAATGG |  |
| BT3SUC-TbMCU-PM-R5-1 | MYTH | ATTCCTGAGAAGAGCAATAAGAAACAGGATCGATTCACCACTGCCCAG |  |
| BT3SUC-TbMCU-PM-F5-2 | MYTH | TGGAACCTGTGTCAGCTGCTCCGGGCAGTGGTGAATCGATCCTGTTTC |  |
| BT3SUC-TbMCU-PM-R5-2 | MYTH | ATCGATTCACCACTGCCCGGAGCAGCTGACACAGGTTCCATGATATCC |  |
| TbMCU-RP-F1 | MYTH | GGTGGCTGGTGGTTGGCTCTGGCTTTGGCCCTTGCGCTGGCTCTTGCTTTATGGTGGGCGGACCTTGACTGGGATATCATGGAACC |  |
| TbMCU-RP-R1 | MYTH | GTCCGCCCACCATAAAGCAAGAGCCAGCGCAAGGGCCAAAGCCAGAGCCAACCACCAGCCACCCGACCACAAGAGTCGCCGCCGCTTCG |  |
| TbMCU-RP-F2 | MYTH | GAAGGCTGGTGGTTGGCTCTGGCTTTGGCCCTTGCGCTGGCTCTTGCTTTATGGTGGGCGAGGAATGGGCGGGTGAAGAGCTAC |  |
| TbMCU-RP-R2 | MYTH | CCTCGCCCACCATAAAGCAAGAGCCAGCGCAAGGGCCAAAGCCAGAGCCAACCACCAGCCTTCCATGATATCCCAGTCAAGGTC |  |
| PR3N-SF | MYTH | GTCGAAAATTCAAGACAAGG |  |
| PR3N-SR | MYTH | AAGCGTGACATAACTAATTAC |  |
| PR3N-TbMCU-F | MYTH | ATTAACAAGGCCATTACGGCCGGGCAGCCGACTTGTAGTGACTC | *Sfi*I |
| PR3N-TbMCU-R | MYTH | AATGATTGGCCGAGGCGGCCTCAGTGATGTTTAAACCACTTCCG | *Sfi*I |
| PR3N-TbMCU-DF1 | MYTH | ATTAACAAGGCCATTACGGCCCTCTTGTGGTCGGGTGCCCTCTG | *Sfi*I |
| PR3N-TbMCU-DR1 | MYTH | AATGATTGGCCGAGGCGGCCTCAGCTCTTCACCCGCCCATTCCTG | S*fi*I |
| BT3SUC-TbMCUb-F | MYTH | ATTAACAAGGCCATTACGGCCTCCAACCCGTACACACCGTTGTG | *Sfi*I |
| BT3SUC-TbMCUb-R | MYTH | AATGATTGGCCGAGGCGGCCCCAGCTCTGTCTTTGGTATCGGAAG | *Sfi*I |
| TbMCUb-NCF1 | MYTH | ATTAACAAGGCCATTACGGCCTTCTTCTGGGCCATTGTTTCCCTC | *Sfi*I |
| BT3SUC-TbMCUb-NCR1 | MYTH | AATGATTGGCCGAGGCGGCCCCGCAGTTCCTCTTGTAGCAAAGAC | *Sfi*I |
| TbMCUb-RP-F1 | MYTH | GCCGGCTGGTGGTTGGCTCTGGCTTTGGCCCTTGCGCTGGCTCTTGCTTTATGGTGGGCGGTTTATGGTTGGAGTGTCATGGAGCC |  |
| TbMCUb-RP-R1 | MYTH | AACCGCCCACCATAAAGCAAGAGCCAGCGCAAGGGCCAAAGCCAGAGCCAACCACCAGCCGGCCCAGAAGAAGCGGCGGCGCTCCATGTC |  |
| TbMCUb-RP-F2 | MYTH | TGCGGCTGGTGGTTGGCTCTGGCTTTGGCCCTTGCGCTGGCTCTTGCTTTATGGTGGGCGTACAAGAGGAACTGCTCATATGAGG |  |
| TbMCUb-RP-R2 | MYTH | GTACGCCCACCATAAAGCAAGAGCCAGCGCAAGGGCCAAAGCCAGAGCCAACCACCAGCCGCAAATAGGCTCCATGACACTCCAACC |  |
| PR3N-TbMCUb-F | MYTH | ATTAACAAGGCCATTACGGCCTCCAACCCGTACACACCGTTGTG | *Sfi*I |
| PR3N-TbMCUb-R | MYTH | AATGATTGGCCGAGGCGGCCTTAAGCTCTGTCTTTGGTATCGGAAG-3’ | *Sfi*I |
| PR3N-TbMCUb-NCR1 | MYTH | AATGATTGGCCGAGGCGGCCTTAGCAGTTCCTCTTGTAGCAAAGAC | *Sfi*I |
| BT3SUC-TbMCUc-F | MYTH | ATTAACAAGGCCATTACGGCCGCATACTGTAGCACTGGAGTCGG | *Sfi*I |
| BT3SUC-TbMCUc-R | MYTH | AATGATTGGCCGAGGCGGCCCCCACCTTTCCGTAGCGAGCAAG | *Sfi*I |
| TbMCUc-NCF1 | MYTH | ATTAACAAGGCCATTACGGCCTTACTGGGCTACTTGATTGTTATATTAC | *Sfi*I |
| BT3SUC-TbMCUc-NCR1 | MYTH | AATGATTGGCCGAGGCGGCCCCGAGGCCCCGAACGGAATAACAATG-3’ | *Sfi*I |
| TbMCUc-RP-F1 | MYTH | TACGGCTGGTGGTTGGCTCTGGCTTTGGCCCTTGCGCTGGCTCTTGCTTTATGGTGGGCGGTCGTTTTTGACTGGAATCTTGTGGAGCC |  |
| TbMCUc-RP-R1 | MYTH | GACCGCCCACCATAAAGCAAGAGCCAGCGCAAGGGCCAAAGCCAGAGCCAACCACCAGCCGTAGCCCAGTAACGGTAGATATAGG |  |
| TbMCUc-RP-F2 | MYTH | 5’ACTGGCTGGTGGTTGGCTCTGGCTTTGGCCCTTGCGCTGGCTCTTGCTTTATGGTGGGCGTCCGTTCGGGGCCTCTCCCTGGAGAC |  |
| TbMCUc-RP-R2 | MYTH | GGACGCCCACCATAAAGCAAGAGCCAGCGCAAGGGCCAAAGCCAGAGCCAACCACCAGCCAGTCATGGGCTCCACAAGATTCC |  |
| PR3N-TbMCUc-F | MYTH | ATTAACAAGGCCATTACGGCCGCATACTGTAGCACTGGAGTCGG | *Sfi*I |
| PR3N-TbMCUc-R | MYTH | AATGATTGGCCGAGGCGGCCCTACACCTTTCCGTAGCGAGCAAG | *Sfi*I |
| PR3N-TbMCUc-NCR1 | MYTH | AATGATTGGCCGAGGCGGCCTTAGAGGCCCCGAACGGAATAACAATG | *Sfi*I |
| BT3SUC-TbMCUd-F | MYTH | ATTAACAAGGCCATTACGGCCCCATTGTGCGTTATTTTGGAACAG | *Sfi*I |
| BT3SUC-TbMCUd-R | MYTH | AATGATTGGCCGAGGCGGCCCCAACACTCTCCAGCCCGCGCAG | *Sfi*I |
| TbMCUd-NCF1 | MYTH | ATTAACAAGGCCATTACGGCCCGCTGCATTGCCATAGCTGTCGCTC | *Sfi*I |
| BT3SUC-TbMCUd-NCR1 | MYTH | AATGATTGGCCGAGGCGGCCCCAGCATCGCTGCTCGTTGCACCGTAC | *Sfi*I |
| TbMCUd-RP-F1 | MYTH | GCCGGCTGGTGGTTGGCTCTGGCTTTGGCCCTTGCGCTGGCTCTTGCTTTATGGTGGGCGTTTCTCTTTGACTGGAACCTCGTGGAGCC |  |
| TbMCUd-RP-R1 | MYTH | AAACGCCCACCATAAAGCAAGAGCCAGCGCAAGGGCCAAAGCCAGAGCCAACCACCAGCCGGCAATGCAGCGATCGGCATAACGCAATCC |  |
| TbMCUd-RP-F2 | MYTH | ACAGGCTGGTGGTTGGCTCTGGCTTTGGCCCTTGCGCTGGCTCTTGCTTTATGGTGGGCGGCAACGAGCAGCGATGCTCGTTTTGAATC |  |
| TbMCUd-RP-R2 | MYTH | TGCCGCCCACCATAAAGCAAGAGCCAGCGCAAGGGCCAAAGCCAGAGCCAACCACCAGCCTGTGATGGGCTCCACGAGGTTCCAGTCAAAG |  |
| PR3N-TbMCUd-F | MYTH | ATTAACAAGGCCATTACGGCCCCATTGTGCGTTATTTTGGAACAG | *Sfi*I |
| PR3N-TbMCUd-R | MYTH | AATGATTGGCCGAGGCGGCCTCAAACACTCTCCAGCCCGCGCAG | *Sfi*I |
| PR3N-TbMCUd-NCR1 | MYTH | AATGATTGGCCGAGGCGGCCTTAAGCATCGCTGCTCGTTGCACCGTAC | *Sfi*I |

The last letter “F”s or “R”s of the primer names stand for “forward” and “reverse” primers, respectively. The underlined nucleotides indicate the introduced restriction endonuclease sites as described in the “enzyme” column. OE stands for overexpression.
