# Supplementary material for: The Mitochondrial Ca2+ Uniporter Complex (MCUC) of Trypanosoma brucei Is a Hetero-oligomer That Contains Novel Subunits Essential for Ca2+ Uptake
Source: mBio. 2018 Sep 18;9(5):e01700-18. doi: 10.1128/mBio.01700-18 (PMC6143741; doi:10.1128/mBio.01700-18)
Supplement: TABLE S2 [file mbo004184060st2.docx]

**Table S2: Plasmids constructed in this study**

| **Application** | **Plasmid name** | **Tag, marker** |
| --- | --- | --- |
|  | pMOTag4mH | smFP-HA, Hyg^R^ |
| ***In situ* smFP-** | pMOTag3mF | smFP-FLAG, G418^R^ |
| **epitope tagging** | pMOTag2mH | smFP-HA, Puro^R^ |
|  | pMOTag2mV | smFP-V5, Puro^R^ |
|  | p2T7 (*TbMCUb*) |  |
| **RNAi** | p2T7 (*TbMCUc*) | Phleo^R^ |
|  | p2T7 (*TbMCUd)* |  |
|  | pLEW100HA-BSD  pLEW100HA-BSD (*TbMCUb*) |  |
| **Overexpression** | pLEW100HA-BSD (*TbMCUc*) | 3×HA, Bsd^R^ |
|  | pLEW100HA-BSD (*TbMCUd*) |  |
|  | pBT3-SUC (*TbMCU*) |  |
|  | pBT3-SUC (*TbMCU∆1*) |  |
|  | pBT3-SUC (*TbMCU∆2*) |  |
|  | pBT3-SUC (*TbMCU∆3*) |  |
|  | pBT3-SUC (*TbMCU∆4*) |  |
|  | pBT3-SUC (*TbMCU∆5*) |  |
|  | pBT3-SUC (*TbMCU∆6*) |  |
|  | pBT3-SUC (*TbMCU∆7*) |  |
|  | pBT3-SUC (*TbMCU∆8*) |  |
|  | pBT3-SUC (*TbMCUb*) | SUC |
|  | pBT3-SUC (*TbMCUb∆1*) | Cub |
| **MYTH (bait)** | pBT3-SUC (*TbMCUb∆2*) | LexA |
|  | pBT3-SUC (*TbMCUb∆3*) | VP16 |
|  | pBT3-SUC (*TbMCUb∆4*) | LEU2 (yeast) |
|  | pBT3-SUC (*TbMCUc*) | Kan^R^ (*E. coli)* |
|  | pBT3-SUC (*TbMCUc∆1*) |  |
|  | pBT3-SUC (*TbMCUc∆2*) |  |
|  | pBT3-SUC (*TbMCUc∆3*) |  |
|  | pBT3-SUC (*TbMCUc∆4)* |  |
|  | pBT3-SUC (*TbMCUd*) |  |
|  | pBT3-SUC (*TbMCUd∆1)* |  |
|  | pBT3-SUC (*TbMCUd∆2)* |  |
|  | pBT3-SUC (*TbMCUd∆3)* |  |
|  | pBT3-SUC (*TbMCUd∆4)* |  |
| **Application** | **Plasmid name** | **Tag,** **Marker** |
|  | pPR3N (*TbMCU*)  pPR3N (*TbMCU∆3*)  pPR3N (*TbMCU∆6*)  pPR3N (*TbMCU∆7*) |  |
|  | pPR3N (*TbMCU∆8*) |  |
|  | pPR3N (*TbMCUb*) |  |
|  | pPR3N (*TbMCUb∆1*) |  |
|  | pPR3N (*TbMCUb∆2*) |  |
|  | pPR3N (*TbMCUb∆3*) |  |
|  | pPR3N (*TbMCUb∆4*) | NubG |
|  | pPR3N (*TbMCUc*) | HA |
| **MYTH (prey)** | pPR3N (*TbMCUc∆1*) | TRP1 (yeast) |
|  | pPR3N (*TbMCUc∆2*) | Amp^R^ (*E. coli)* |
|  | pPR3N (*TbMCUc∆3*) |  |
|  | pPR3N (*TbMCUc∆4*) |  |
|  | pPR3N (*TbMCUd*) |  |
|  | pPR3N (*TbMCUd∆1*) |  |
|  | pPR3N (*TbMCUd∆2*) |  |
|  | pPR3N (*TbMCUd∆3*) |  |
|  | pPR3N (*TbMCUd∆4*) |  |
| Abbreviations: smFP, “spaghetti monster” fluorescent protein; HA, hemagglutinin; R, resistance; Hyg, hygromycin; Puro, puromycin; Phleo, phleomycin; Bsd, blasticidin; *∆,* mutation*;* Kan, kanamycin; SUC, *SUC2* signal sequence derived from the yeast invertase protein; Cub, C-terminal half of ubiquitin; LexA, the *E. coli* LexA protein; VP16, the *Herpes simplex* VP16 transctivator protein; LEU2, *LEU2* auxotrophic marker for selection in yeast; NubG, N-terminal half of ubiquitin with an isoleucine (I) to glycine (G) exchange at position 13 of the Nub moiety; TRP1, *TRP1* auxotrophic marker for selection in yeast; Amp, ampicillin. | | |
|  |  |  |
|  |  |  |
