# Supplementary material for: The Mitochondrial Ca2+ Uniporter Complex (MCUC) of Trypanosoma brucei Is a Hetero-oligomer That Contains Novel Subunits Essential for Ca2+ Uptake
Source: mBio. 2018 Sep 18;9(5):e01700-18. doi: 10.1128/mBio.01700-18 (PMC6143741; doi:10.1128/mBio.01700-18)
Supplement: TABLE S3 [file mbo004184060st3.docx]

**Table S3. Antibodies used in this study**

| **Antibody name** | **Antigen** | **Raised in** | **Source** | **Catalogue no.**  **[Reference]** | **IFA dilution** | **WB dilution** |
| --- | --- | --- | --- | --- | --- | --- |
| Anti-FLAG | FLAG peptide-tag  (DYKDDDDK) | Rabbit | Sigma | F7425 | 1:1,000 | 1:20,000 |
| Anti-HA | HA peptide-tag  (YPYDVPDYA) | Mouse | Covance | MMS-101P | 1:50 | 1:1,000 |
| Anti-HA | HA peptide-tag  (YPYDVPDYA) | Rabbit | Abcam | Ab9110 | 1:1,000 | 1:20,000 |
| Anti-hexokinase | Yeast hexokinase | Rabbit | Rockland | 200-4159S |  | 1:20,000 |
| Anti-TbCyt c1 | *T. brucei* cytochrome c1 | Rabbit | Dr. S. Hajduk | [1] |  | 1:5,000 |
| Anti-TbMCU | *T. brucei* MCU | Mouse | This lab | [2] | 1:100 | 1:1,000 |
| Anti-TbVDAC | *T. brucei* VDAC | Rabbit | Dr. M. Chaudhuri | [3] |  | 1:2,000 |
| Anti-V5 | V5 peptide-tag  (GKPIPNPLLGLDST) | Mouse | Thermo Fisher | R960-25 | 1:100 | 1:2,500 |
| Anti-V5 | V5 peptide-tag  (GKPIPNPLLGLDST) | Rabbit | Sigma | V8137 | 1:500 |  |
| Anti-VP16 | *Herpes simplex* virus VP16 | Rabbit | Sigma | V4388 | 1:100 | 1:1,000 |
| Anti-tubulin | Human alpha-tubulin | Rabbit | Sigma | SAB3501071 |  | 1:10,000 |
| GAM 488  (2^nd^ antibody) | Mouse IgG (H+L), Alexa Fluor 488 conjugate | Goat | Thermo Fisher | A-11029 | 1:1,000 |  |
| GAR 488  (2^nd^ antibody) | Rabbit IgG (H+L), Alexa Fluor 488 conjugate | Goat | Thermo Fisher | A-11034 | 1:1,000 |  |
| GAM 546  (2^nd^ antibody) | Mouse IgG (H+L), Alexa Fluor 546 conjugate | Goat | Thermo Fisher | A-11030 | 1:1,000 |  |
| GAR 546  (2^nd^ antibody) | Rabbit IgG (H+L), Alexa Fluor 546 conjugate | Goat | Thermo Fisher | A-11035 | 1:1,000 |  |
| GAM-HPR  (2^nd^ antibody) | Mouse IgG (H+L), HPR conjugate | Goat | Bio-Rad | 172-1011 |  | 1:15,000 |
| GAR-HPR  (2^nd^ antibody) | Rabbit IgG (H+L), HPR conjugate | Goat | Bio-Rad | 170-6515 |  | 1:15,000 |

Abbreviations: IFA, immunofluorescence assay; WB, Western blot analysis; HA, hemagglutinin; GAM, goat anti-mouse; GAR, goat anti-rabbit; HPR, horseradish peroxidase; VDAC, voltage-dependent anion channel

[1] J. W. Priest, S. L. Hajduk, *Trypanosoma brucei* cytochrome c1 is imported into mitochondria along an unusual pathway, The Journal of biological chemistry 278 (2003) 15084-15094.

[2] G. Huang, A.E. Vercesi, R. Docampo, Essential regulation of cell bioenergetics in *Trypanosoma brucei* by the mitochondrial calcium uniporter, Nat Communl 4l (2013) 2865.

[3] U. K. Singha, S. Sharma, M. Chaudhuri, Downregulation of mitochondrial porin inhibits cell growth and alters respiratory phenotype in *Trypanosoma brucei,* 8 *(9)* Eukaryotic Cell (2009) 1418-1428.
